# Engineering osmolysis susceptibility in *Cupriavidus necator* and *Escherichia coli* for recovery of intracellular products

**DOI:** 10.1186/s12934-023-02064-8

**Published:** 2023-04-12

**Authors:** Jeremy David Adams, Kyle B. Sander, Craig S. Criddle, Adam P. Arkin, Douglas S. Clark

**Affiliations:** 1 Department of Chemical and Biomolecular Engineering, University of California, Berkeley, CA 94720 USA; 2 Department of Bioengineering, University of California, Berkeley, CA 94720 USA; 3 Department of Civil and Environmental Engineering, Stanford University, Stanford, CA 94305 USA; 4 Environmental Genomics and Systems Biology Division, Lawrence Berkeley National Laboratory, 1 Cyclotron Road, Berkeley, CA 94720 USA; 5 Molecular Biophysics and Integrated Bioimaging Division, Lawrence Berkeley National Laboratory, 1 Cyclotron Road, Berkeley, CA 94720 USA

**Keywords:** Osmolysis, Bioseparations, Bacteria cell lysis, Adaptive laboratory evolution, Mechanosensitive channel

## Abstract

**Background:**

Intracellular biomacromolecules, such as industrial enzymes and biopolymers, represent an important class of bio-derived products obtained from bacterial hosts. A common key step in the downstream separation of these biomolecules is lysis of the bacterial cell wall to effect release of cytoplasmic contents. Cell lysis is typically achieved either through mechanical disruption or reagent-based methods, which introduce issues of energy demand, material needs, high costs, and scaling problems. Osmolysis, a cell lysis method that relies on hypoosmotic downshock upon resuspension of cells in distilled water, has been applied for bioseparation of intracellular products from extreme halophiles and mammalian cells. However, most industrial bacterial strains are non-halotolerant and relatively resistant to hypoosmotic cell lysis.

**Results:**

To overcome this limitation, we developed two strategies to increase the susceptibility of non-halotolerant hosts to osmolysis using *Cupriavidus necator*, a strain often used in electromicrobial production, as a prototypical strain. In one strategy, *C. necator* was evolved to increase its halotolerance from 1.5% to 3.25% (w/v) NaCl through adaptive laboratory evolution, and genes potentially responsible for this phenotypic change were identified by whole genome sequencing. The evolved halotolerant strain experienced an osmolytic efficiency of 47% in distilled water following growth in 3% (w/v) NaCl. In a second strategy, the cells were made susceptible to osmolysis by knocking out the large-conductance mechanosensitive channel (*mscL*) gene in *C. necator*. When these strategies were combined by knocking out the *mscL* gene from the evolved halotolerant strain, greater than 90% osmolytic efficiency was observed upon osmotic downshock. A modified version of this strategy was applied to *E. coli* BL21 by deleting the *mscL* and *mscS* (small-conductance mechanosensitive channel) genes. When grown in medium with 4% NaCl and subsequently resuspended in distilled water, this engineered strain experienced 75% cell lysis, although decreases in cell growth rate due to higher salt concentrations were observed.

**Conclusions:**

Our strategy is shown to be a simple and effective way to lyse cells for the purification of intracellular biomacromolecules and may be applicable in many bacteria used for bioproduction.

**Supplementary Information:**

The online version contains supplementary material available at 10.1186/s12934-023-02064-8.

## Background

Whole-cell biocatalysis encompasses a wide range of existing and potential processes in which microbes convert low-value feedstocks to higher-value products. Biochemical processes can produce biomolecules such as proteins that cannot be produced by traditional chemical processes, as well as fuels, commodity chemicals, and bioplastics that would otherwise be produced in petrochemical processes that contribute to anthropogenic climate change [1–4]. Downstream separations of the desired product are an important and costly component of any bioprocess [5] and can vary significantly depending on whether the product of interest is extracellular or intracellular. Certain industrial microbial hosts, for example *Bacillus subtilis* and *Bacillus licheniformis*, secrete enzymes such as proteases with high yields to the extracellular space, which allows for relatively simple purification of these biomolecules [6]. However, such strategies are limited to specific proteins produced in certain strains, as not all biomolecular products are suited for transport across the cell membrane. Intracellular macromolecular bioproducts on the other hand can be challenging to separate from bacterial biomass as these molecules cannot easily diffuse through the cell membrane, and therefore require cellular disruption to recover the product.

Yet, intracellular macromolecules represent an important class of bioproducts. For example, recombinant proteins (industrial enzymes and biopharmaceuticals, in particular) are widely used intracellular products [7]. Moreover, the demand for high-quality plasmid DNA, generally produced as an intracellular product in bacteria such as *E. coli*, has greatly increased as more cell and gene therapies have been developed [8]. Certain bioplastics such as polyhydroxyalkanoates (PHAs) are produced as full-length polymers in many bacteria [4]. PHAs, most notably polyhydroxybutyrate (PHB), are native products in many bacteria that use them as a store of carbon and energy [9, 10]. Non-native PHB producers, such as *E. coli*, have also been engineered for PHB production [11].

Traditional bioseparations of biomacromolecules first require cell lysis prior to downstream purification of the desired product [12]. Mechanical methods such as ultrasonication and high-pressure homogenization can efficiently lyse cells, though these require expensive equipment, are energy-intensive, and may damage sensitive biomolecules [12, 13]. Chemical and enzymatic methods of cell lysis can also be used to liberate intracellular products, though the cost of the materials make these techniques difficult to scale [14, 15]. Novel methods of lysing bacterial cells that are cheap and simple are therefore desirable.

**Fig. 1:**
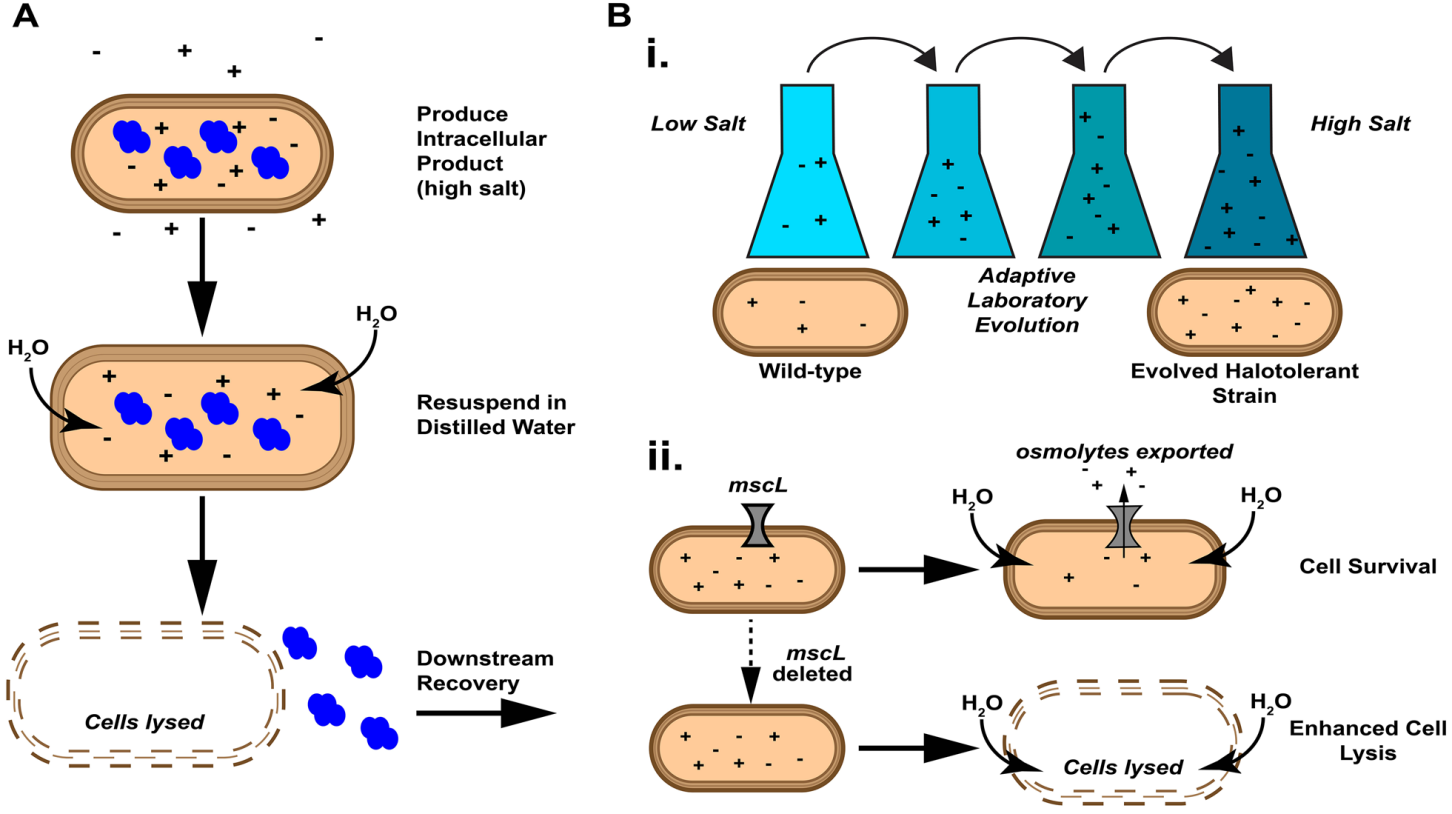
Overview of approach. **A** Schematic representation of osmolysis-based recovery of intracellular biomacromolecules. Product is first produced by microbial host under elevated salt concentrations. Cells are then resuspended in distilled water, causing an increase of turgor pressure due to osmotic shock, which lyses the cell membrane and enables downstream recovery of the product. **B** Two orthogonal strategies employed here to increase the sensitivity of microbial hosts to osmotic downshock. In one strategy (i) ALE is used to increase the halotolerance of the microbe, enabling cell growth at higher salt concentrations and therefore a greater magnitude of osmotic downshock when cells are resuspended in distilled water. In the other strategy (ii) the large-conductance mechanosensitive channel (*mscL*) or a related gene is deleted from the microbial host, which limits the ability of cells to export osmolytes in hypotonic solutions, increasing their susceptibility to osmotic lysis

Osmolysis is a simple, low-cost method of cell lysis that relies on osmotic pressure to swell cells and burst membranes following the resuspension of cells in a hypotonic solution (Fig. 1A). Osmolysis as a cell lysis technique in downstream separations has traditionally been restricted to mammalian cell culture, where the weaker cell membrane is fairly labile to osmotic pressure changes [14]. The more robust bacterial cell wall, as well as stress-response survival mechanisms, allow most bacteria to survive moderate fluctuations of osmolarity [14, 16]. More recently, extremely halophilic bacteria have been explored as microbial PHB producers [17, 18]. Extreme halophiles can grow in salinities from 15 to 30% NaCl (w/v) [19], and therefore resuspension of these microbes in distilled water will cause a much higher osmotic pressure shock than can be achieved with bacteria grown in conventional media. For example, Rathi et al*.* demonstrated a PHB recovery of 98% from pre-dried *Halomonas* sp. SK5 biomass using this technique [17]. Osmolysis is therefore a promising technique to reduce the energy demand, material use, and cost of bioseparations. However, extremely halophilic bacteria are rarely, if at all, used in industrial bioprocesses, and many applications call for specific bacterial strains that are likely not halophilic.

Electromicrobial production (EMP) is an emerging technology with the potential to generate a wide array of useful bioproducts. EMP relies on bacteria that utilize electricity or electrochemically generated mediator molecules such as hydrogen gas and formic acid as energy sources to produce various bioproducts [20]. Traditional biochemical systems use crop-derived sugars as microbial substrates and therefore cause social and environmental impacts such as carbon emissions from fertilizer production, nitrous oxide emissions from fertilizer application, land use effects, and competition with the food supply [21, 22]. EMP systems, however, do not rely on the agricultural system, and, if using a clean electricity source, can lead to a decreased global warming potential and land occupation footprint [23].

A particularly promising microbe studied for EMP systems is *Cupriavidus necator*, a soil bacterium capable of growth on various substrates, including H_2_/CO_2_, formate, and organic molecules [24]. Electrolysis of water to produce hydrogen or electrochemical reduction of carbon dioxide to formic acid can therefore be used to generate substrates that Knallgas or formatotrophic bacteria (both of which describe *C. necator*) can convert to desired products [25, 26]. *C. necator* naturally produces the polyester PHB, and is often regarded as a model organism for PHA production due to its ability to accumulate high levels of the polymer intracellularly (up to 90% of total cell mass) and its potential in producing many PHA variants [27, 28]. In addition, expression systems have been developed for *C. necator* that allow production of recombinant proteins [29, 30], and metabolic engineering has been applied to produce various fuels and commodity chemicals such as isopropanol, acetoin, and various alkanes [31, 32].

While EMP addresses the environmental impacts of substrate generation in bioprocessing, a sustainable bioproduction system must also minimize energy and resource demand during separations. Adapting the osmolysis cell disruption method to work with EMP-relevant microbes could address the resource-intensive separations process for intracellular macromolecular products produced through EMP. However, to our knowledge, no EMP systems have used halophilic or halotolerant bacteria. We address this issue by developing a two-part strategy to render non-halophilic bacteria susceptible to lysis by osmotic downshock, using *C. necator*, a microbe commonly used in EMP systems as a model host (Fig. 1B). We demonstrate that intracellular biomolecule products can then be separated from the cells, using recombinant red fluorescent protein (RFP) as a useful example product due to its ease of measurement.

We first use adaptive laboratory evolution (ALE) to improve the halotolerance of *C. necator*, which enables a greater magnitude of osmolarity change and therefore greater osmotic pressure when the cells are resuspended in distilled water. In parallel, we rationally engineer *C. necator* by knocking out the large-conductance mechanosensitive channel (*mscL*) gene, a membrane protein that facilitates cell survival during hypotonic shock that is found in a wide range of bacteria [33, 34]. While either method individually can improve the susceptibility of the bacteria to osmolysis, we then show that combining these two methods in a single strain (*i.e.*, one that is both halotolerant and lacks the *mscL* gene) enables significantly higher osmolytic efficiency than either method individually. To measure the osmolytic efficiency of cells lysed upon osmotic downshock, we develop an RFP-based assay to determine the fraction of intracellular contents released to the media. We finally demonstrate that this approach can be expanded to other bacteria by adapting the approach to *E. coli* BL21, a strain routinely used in the production of recombinant proteins. Both the *mscL* gene, and the related small-conductance mechanosensitive channel (*mscS*) gene are knocked out of BL21 to make it susceptible to osmolysis.

## Methods

### Microbial media and culturing methods

All *E. coli* strains were grown at 37 °C and all *C. necator* strains were grown at 30 °C unless otherwise stated. Luria Broth (LB) was used as media for all *E. coli* cultures unless otherwise stated. Media were supplemented with kanamycin (50 μg/mL *E. coli*, 200 μg/mL *C. necator*) and/or carbenicillin (100 μg/mL) as appropriate. All liquid cultures were shaken at 200 RPM. All bacterial strains used in the study (for list, see Table 1) were stored at −80 °C in 25% glycerol until needed. To reactivate the strains, cells were first streaked onto agar plates (with appropriate antibiotic as needed), incubated, and then a liquid LB culture was started from a single colony.

**Table 1:** Strains and plasmids used in this study

Strain/plasmid	Description	Source
Strains		
*C. necator* H16	Wild-type *Cupriavidus necator strain*	DSM 428
*C. necator* H16 *ΔmscL*	*C. necator* strain deficient in gene encoding large-conductance mechanosensitive channel	This work
*C. necator* ht030b	*C. necator* strain with improved halotolerance following 250 generations of adaptive laboratory evolution	This work
*C. necator* ht030b *ΔmscL*	Adapted halotolerant *C. necator* strain deficient in gene encoding large-conductance mechanosensitive channel	This work
*E. coli* WM3064	DAP-auxotrophic *E. coli* donor strain used for conjugation of *C. necator*	William Metcalf (UIUC)
*E. coli* BL21	*E. coli* B strain deficient in Lon and OmpT proteases widely used in protein expression	New England Biolabs
*E. coli* BL21 *ΔmscL*	*E. coli* BL21-derived strain deficient in gene encoding large-conductance mechanosensitive channel	This work
*E. coli* BL21 *ΔmscL ΔmscS*	*E. coli* BL21-derived strain deficient in genes encoding large-conductance mechanosensitive channel and small-conductance mechanosensitive channel	This work
Plasmids		
pBADTrfp	araBAD promoter, T7-stem loop, Kan^R^, mRFP1 expression gene. Addgene #99382	[29]
pMQ30k	pMQ-30 derivative with Kan^R^ marker; SacB sucrose sensitivity gene; integrating plasmid used for gene deletion in *C. necator*	[35]
pMQ30k-ΔmscL	pMQ30k plasmid derivative containing 500 nt upstream and 500 nt downstream of *mscL* gene	This work
pSIJ8	Temperature-sensitive plasmid expressing lambda Red recombinase and flippase recombinase genes; Amp^R^; for gene deletion in *E. coli*. Addgene #68122	[36]

### Adaptive laboratory evolution

The halotolerance of *Cupriavidus necator* was improved by adaptive laboratory evolution (ALE) in 10-mL batch cultures. Wild-type *C. necator* H16 was grown in 50-mL tubes with 10 mL LB medium with NaCl starting at 15 g/L (final concentration). After 24 h of growth, cells were passaged into 10 mL fresh LB medium in 50-mL tubes at an initial optical density of A_600_ = 0.001. At the end of each passage, the average growth rate was calculated from the initial and final culture densities, assuming constant exponential growth. When the average growth rate either plateaued or exceeded 0.3 h^−1^, the salt concentration of the culture was increased by 0.25% (w/v) NaCl. This was repeated for 30 passages. Cells were plated every several passages on LB Agar plates to ensure the cultures were free of contamination. The final passage was plated on LB Agar with 3% NaCl (w/v, final concentration) and a single colony was selected for further experiments, with the strain named *C. necator* ht030b.

The growth rate of strain ht030b was compared to that of wild-type *C. necator* H16 at elevated salt concentrations. Overnight cultures of each respective strain were inoculated into four 1-mL volumes of LB supplemented with 3% NaCl (w/v, final concentration) in a 24-well plate at a cell density of A_600_ = 0.01 and grown overnight at 30 °C. Absorbance measurements (600 nm) were taken every hour.

### Genomic methods

The final strain from the adaptive laboratory evolution, ht030b, was streaked on an LB plate containing 3% (w/v) NaCl. Two colonies were grown overnight in liquid LB containing 3% (w/v) NaCl, and the genome was purified from each sample using a Monarch^®^ Genomic Purification Kit (New England Biolabs). A single colony of wild-type H16 was grown, and the genome was likewise purified as a control. Extracted genomic DNA was provided to the Vincent J. Coates Genomics Sequencing Lab at the University of California, Berkeley (QB3 Genomics, UC Berkeley, Berkeley, CA, RRID:SCR_022170), which prepared 150 bp paired-end Illumina sequencing libraries. These libraries were then sequenced using a NovaSeq 6000 S4 Sequencing System. Single nucleotide polymorphisms and other genomic variants were determined using requisite applications within the Geneious Prime software (version 2022.2.2, https://www.geneious.com). SNP’s/variants were called from mapped reads originating from three different samples: one sample of the unevolved, wildtype *C. necator* H16 strain, which served as input to the adaptive laboratory evolution, and two different samples of the evolved strain exhibiting elevated halotolerance.

Paired reads were first trimmed to remove low quality bases, filter out and remove short reads (< 10 bp), remove sequencing adapter content, and trim/remove low complexity regions using the ‘Trim using BBDuk’ application within the software. The ‘minimum quality’ setting was set to 30 and all other parameters were left at their default values. Reads were then mapped to a *C. necator* H16 reference genome composed of GenBank accession numbers CP039287, CP039288, and CP039289 [37]. The application ‘Map to Reference’ was used for this process with the Sensitivity set to ‘Medium–Low Sensitivity-Fast’ and all other parameters set to their default values. The application ‘Find Variations/SNP’s’ was then used to identify variations within the mapped assemblies with all parameters set to their default values. Identified variants were then manually filtered to remove those that were not represented with at least 27X coverage, variant frequencies < 90% among mapped reads covering the candidate variant position, and those exhibiting a strand bias < 25% or > 75%. Tandem repeat variants > 5 bp were also filtered out and not considered further.

Variants were first called from reads originating from the unevolved *C. necator* H16 sample in order to identify differences between this assembly and the reference genome. Variations found to be unique to either of the evolved populations and not present in the unevolved *C. necator* H16 sample assembly, and meeting aforementioned criteria, were considered. Also considered were variations that were found in the *C. necator* H16 unevolved samples but not found in the evolved populations.

### Transformation of plasmids to *Cupriavidus necator*

*C. necator* strains were transformed with plasmids in a two-step method in which the plasmids were first transformed to chemically competent *E. coli* WM3064 cells by heat shock, followed by conjugation of the plasmid from the WM3064 donor strain to *C. necator*. Strain WM3064 was a gift from William Metcalf (University of Illinois). Chemically competent WM3064 cells were made as follows. WM3064 cells were cultured in LB containing diaminopimelic acid (DAP, 0.3 μM) to an optical density of A_600_ = 0.4. Cells were chilled on ice for 20 min before being pelleted via centrifugation at 4,000 g for 10 min. Cells were resuspended in ice cold 100 mM CaCl_2_ solution and incubated on ice for one hour. Cells were then centrifuged at 4,000 g and resuspended in 100 mM CaCl_2_ solution with 10% glycerol at 50 × the original cell concentration. Cells were stored in 50 μL aliquots at -80 °C until needed.

All *C. necator* strains listed in Table 1 were transformed with the red fluorescent protein (RFP) expression plasmid pBADTrfp via conjugation using *E. coli* WM3064 as a donor, following the protocol from Windhorst et al*.* [31]. pBADTrfp was a gift from Nathan Hillson (Addgene plasmid # 99382; http://n2t.net/addgene:99382; RRID:Addgene_99382) [29]. pBADTrfp was transformed into chemically competent WM3064 cells using heat shock and positive clones were selected for on LB-Agar plates containing DAP and kanamycin (50 μg/mL) following a 1-h outgrowth in SOC medium. The *C. necator* strain and the transformed WM3064 strain were both grown overnight in LB and LB with DAP and kanamycin, respectively. The following day, the two cultures were inoculated into a fresh culture of the same media and were allowed to grow until the optical density of each culture reached A_600_ ~ 0.5. The WM3064 culture was washed twice with LB DAP, and 0.75 mL of each culture was mixed. This mixture was pelleted by centrifugation at 8,000 g for 10 min, resuspended in 100 μL of LB-DAP, plated on a nitrocellulose membrane on an LB DAP plate, and incubated at 30 °C for 18 h. The filter was then resuspended in 2 mL of LB with kanamycin (200 μg/mL) and 50 μL was plated on an LB agar plate with kanamycin (200 μg/mL). The conjugation plate was incubated at 30 °C for 2 days. Proper transformation of the plasmid was confirmed via colony PCR.

### Gene deletion in *Cupriavidus necator*

Strains lacking the *mscL* gene were generated following a method relying on integrative plasmids and sucrose counterselection adapted from Windhorst et al*.* [31]. A gene fragment containing a 500-nucleotide region matching the region upstream of the *mscL* gene in *C. necator* H16 followed by a 500-nucleotide matching the region downstream was synthesized by IDT DNA Technologies. Overhang regions matching 20 nucleotide-long regions upstream and downstream of EcoRI and BamHI cut sites, respectively, were added by PCR, and the fragment was assembled with the linearized pMQ30k vector by Gibson Assembly [38], yielding the plasmid pMQ30k-ΔmscL. This plasmid was transformed into *C. necator* H16 and ht030b via conjugation with WM3064 as described above, and cells were selected on an LB agar plate with kanamycin (200 μg/mL). Kanamycin-resistant colonies were then grown overnight in liquid LB supplemented with kanamycin (200 μg/mL). This culture was then passaged in a 1000-fold dilution into LB without antibiotics and cultured for 24 h. The cells were plated on an LB agar plate supplemented with 15% (w/v) sucrose for counterselection. Proper gene deletion was confirmed by colony PCR and Sanger Sequencing.

### Gene deletion in* E. coli BL21*

The *mscL* gene was deleted from *E. coli* BL21 using a lambda Red recombination system as has been established previously [36]. A gene cassette, containing a kanamycin resistance gene flanked by a flippase recognition target (FRT) site on each end, and with homology arms matching 120 bp upstream and downstream of the *E. coli* BL21 *mscL* gene on the 5’- and 3’- termini, was synthesized by Integrated DNA Technologies. Chemically competent BL21 cells (New England Biolabs) were transformed with pSIJ8, a temperature-sensitive plasmid that contains arabinose-inducible λ-Red recombinase genes and rhamnose-inducible flippase recombinase gene. pSIJ8 was a gift from Alex Nielsen (Addgene plasmid # 68122; http://n2t.net/addgene:68122; RRID:Addgene_68122) [36]. Strains containing the pSIJ8 plasmid were grown at 30 °C to maintain plasmid stability. BL21 pSIJ8 was grown in 15 mL of Terrific Broth (TB) medium (supplemented with carbenicillin) until reaching a cell density of A_600_ = 0.35, followed by a 45-min induction with arabinose (2 mg/mL, final concentration). Cells were then made electrocompetent by four consecutive wash steps in chilled 10% (v/v) glycerol solution, which concentrated cells ~ 100-fold. On ice, 5 μL (250 ng) of the synthetic DNA cassette were added to 50 μL of electrocompetent cells and cells were electroporated (1.8 kV, 1 mm gap, BTX Gemini X2). Cells were recovered with 950 μL TB for 3 h. Cells from the outgrowth were pelleted and resuspended in 200 μL TB, and cells were plated on LB Agar plates with kanamycin and carbenicillin and grown for 36 h. A single colony was selected and grown in LB with kanamycin and carbenicillin overnight. The following day, the culture was washed in LB, diluted to a cell density of A_600_ = 0.1, and flippase expression was induced for 4 h with 50 mM L-rhamnose, which removed the integrated kanamycin gene from the BL21 genome. Serial dilutions were performed and cells were plated on LB Agar with carbenicillin and grown overnight. Correct gene deletions were verified by colony PCR. This strain was saved for further experiments. The *mscS* gene was then deleted from the BL21 *ΔmscL* strain using a similar DNA cassette with homology arms matching 120 bp upstream and downstream of the *E. coli* BL21 *mscS* gene, following the same protocol. The plasmid pSIJ8 was then cured from both BL21 *ΔmscL ΔmscS* and BL21 *ΔmscL* by growing them overnight in LB at 37 °C without antibiotics.

### RFP-Based lysis assay for *C. necator* cells

An RFP-based lysis assay was developed to measure the osmolysis efficiency, or the fraction of cells that lysed upon resuspension in distilled water. The *C. necator* strain of interest was first transformed with the expression vector pBADTrfp, which contains an arabinose-inducible RFP gene, by conjugation. The *C. necator* strain carrying pBADTrfp was grown overnight in LB (final NaCl concentration 1.5% for the non-halotolerant strain and 3.0% for the evolved strain). For experiments relying on heterotrophic growth, cells from the overnight culture were inoculated into LB (at appropriate salt concentration). In mid-exponential phase (A_600_ ~ 0.5), cells were induced with arabinose (final concentration 1 mg/mL) and RFP was expressed overnight at room temperature. For experiments relying on organoautotrophic growth, cells from the overnight culture (grown in LB) were inoculated into M9 minimal salts medium supplemented with 4 g/L sodium formate and either 6 g/L NaCl (for the non-halotolerant strain) or 16 g/L NaCl (for the halotolerant strain). Cells were grown overnight, pelleted, and resuspended in fresh medium containing arabinose (final concentration 1 mg/mL). RFP was expressed overnight at room temperature.

For both heterotrophic and organoautotrophic experiments, following overnight expression, the cells were washed once in their respective growth media and the cell concentration was adjusted to A_600_ = 1.0. Cells were aliquoted in 1-mL volumes and centrifuged at 4,000 g for 10 min. Cells were resupended in an aqueous solution containing various NaCl concentrations representing either an isotonic or hypotonic solution, and were shaken at 30 °C for 30 min. Two 150-μL samples were taken from the well-mixed cell solution and the fluorescence intensity was measured by a Tecan Spark^®^ 10 M microplate reader (Ex. 585 nm/ Em. 620 nm). Samples were diluted as needed to ensure measurements fell within the linear range (Additional file 1: Fig. S4). The rest of the cell solution was centrifuged at 4,000 g for 10 min and two 150-μL samples of the supernatant were collected and their fluorescent signal was measured as before in order to quantify the amount of RFP released to the extracellular space. The ratio of fluorescent signal in the supernatant and cell solutions was taken to be the fraction of cells lysed.

### RFP-Based lysis assay for *E. coli* BL21 cells

*E. coli* strains BL21 *ΔmscL* and BL21 *ΔmscL ΔmscS* were made chemically competent following the same protocol as for WM3064, except for the omission of DAP. Chemically competent BL21 *ΔmscL ΔmscS*, BL21 *ΔmscL*, and wild-type BL21 cells were transformed with pBADTrfp via heat shock and selected for on LB Agar plates with kanamycin (50 μg/mL). Osmolysis experiments were performed following the same protocol as for *C. necator*, with minor adjustments. Overnight cultures were regrown at 37 °C in LB supplemented with the appropriate NaCl concentration. In mid-exponential phase (A_600_ ~ 0.5), cells were induced with arabinose (final concentration 1 mg/mL) and RFP was expressed for 3 h at 30 °C. The rest of the osmolysis assay follows the exact protocol as for *C. necator*. Samples from the total cell fraction, post-lysis supernatant, and cell pellet were saved for SDS-PAGE.

## Results and discussion

### Adaptive laboratory evolution of *C. necator* improves halotolerance

**Fig. 2:**
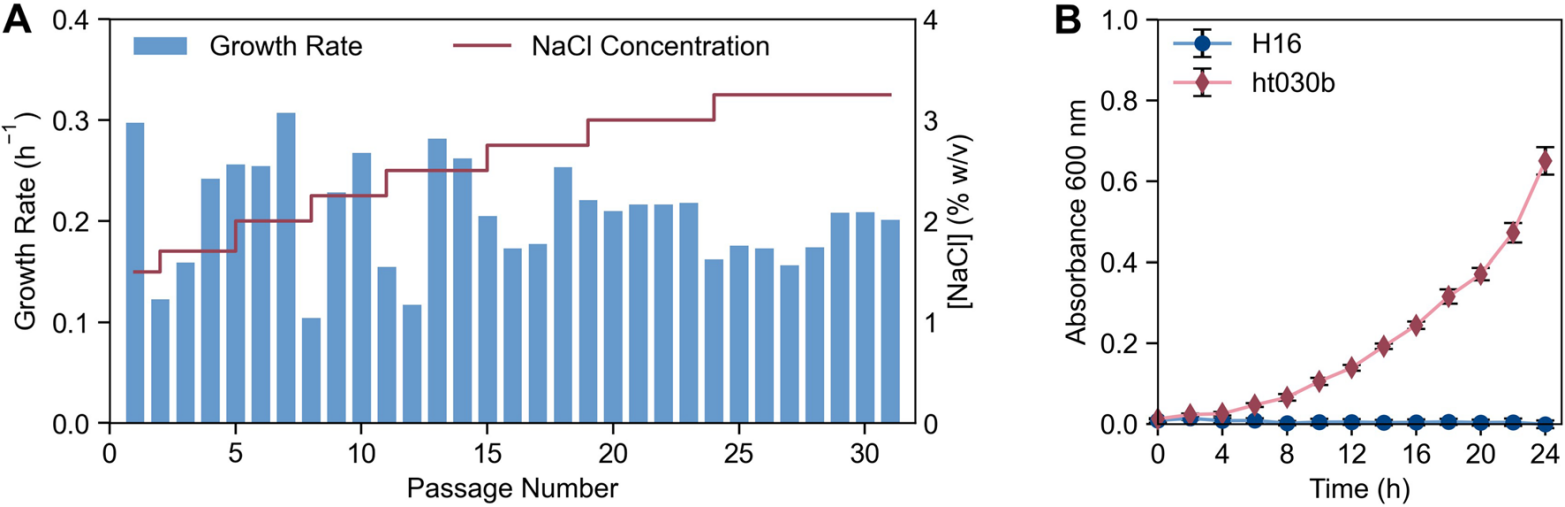
Adaptive laboratory evolution yields halotolerant strain of *C. necator*. **A** Results of ALE experiment showing the growth rate (blue bars) and NaCl concentration (red line) at each passage. **B** Growth curve of wild-type *C. necator* strain H16 and evolved strain ht030b in LB containing 3% NaCl (w/v, final concentration). For each data point in (**B**), mean ± SD is displayed (n = 4)

Through 30 passages of ALE, accounting for roughly 250 generations of growth, the tolerance of *C. necator* in NaCl was improved from 1.5% to 3.25% (Fig. 2a). Microbial behavior during the ALE process was typical compared to studies performed previously. Sharp decreases in growth rate following addition of the stress (in this case NaCl) were regularly observed. Similar to other ALE experiments [39, 40], the steepest increases in cell fitness were observed in early passages, with growth rates mostly plateauing in later passages. For example, the NaCl tolerance of *C. necator* improved by 1.25% (from 1.5% to 2.75%) in the first 15 passages, while only improving an additional 0.5% in the later fifteen passages. The osmolarity of 3.25% (w/v) NaCl, where the ALE began to plateau, is nearly identical to that of seawater. Given the existence of extremely halophilic proteobacteria such as *Halomonas* sp., it is plausible that the halotolerance of *C. necator* could improve with further rounds of ALE. However, it is likely these adaptations would occur significantly more slowly than the initial improvements observed here.

The growth of evolved strain ht030b, in comparison to the wild-type H16 strain, was tested in LB containing a (final) concentration of 3.25% NaCl (Fig. 2b). Under the conditions tested, the wild-type H16 did not display measurable growth over a 24-h period while strain ht030b grew with a growth rate of 0.16 h^−1^. We do note that when seeded with a high enough starting cell concentration, *C. necator* H16 displays some growth in LB with 3.25% NaCl, albeit at a significantly lower rate as compared to ht030b (Additional file 1: Fig. S1). In any case, the strain generated through the ALE displays a clear phenotypic difference from the wild-type strain. This growth rate is significantly lower than the growth rate of wild-type *C. necator* in LB containing the standard 0.5% NaCl (0.45 h^−1^). Therefore, despite the improved fitness compared to the wild-type, strain ht030b grows more slowly as salt concentration is increased, introducing a tradeoff between higher media salinity and growth rate.

### Genomic analysis of ht030b

Genomic sequencing and the subsequent variant analysis identified five mutations (meeting the filtering criteria applied) that were acquired by strain ht030b throughout the course of the ALE (Additional file 1: Table S1). Four mutations were present only in the ht030b genome, but not in either the reference H16 genome used for mapping or in the unevolved H16 genome sequenced. Each caused substitutions of a single amino acid in a different protein: a PAS domain-containing sensor histidine kinase *NtrY*, a peptidoglycan D,D-transpeptidase *mrdA*, an acetolactate synthase gene, and an IS66 family transposase gene. One SNP, causing a point substitution in a *YgcG* family protein, was surprisingly found only in the H16 genome, but not in the evolved strain ht030b or in the reference genome. This indicates that this mutation was present in the starting H16 strain but reverted to the original sequence as found in the reference genome throughout the course of the ALE. Unfortunately, the function of this gene family is unknown (although it is predicted to have transmembrane domains), limiting our understanding of the mechanism by which this could affect halotolerance.

The *mrdA* gene encodes an enzyme in the penicillin-binding protein 2 (PBP2) family, and is involved in the synthesis in the peptidoglycan cell wall in bacteria, particularly in cell elongation [41]. Penicillin-binding proteins have been shown to be important to cell survival under conditions of high salt stress, perhaps in response to the morphological changes (*i.e.* cell plasmolysis) that occur during osmotic upshifts [42, 43]. Hocking et al*.* demonstrated recruitment of an *mrdA* homolog to the cell division site in *Caulobacter crescentus* in response to increases in salinity [44], suggesting the regulation of this gene is indeed involved in the osmotic stress-response mechanism. Therefore, it is conceivable that a change in either expression level or activity due to the detected mutation in the *mrdA* gene causes structural changes of the cell wall in *C. necator* ht030b, leading to enhanced survival in high-salt conditions. Interestingly, mutations of this type seem to be highly conserved in bacterial species. Strain ht030b displays a T563I mutation, substituting the polar amino acid threonine with a branched chain nonpolar amino acid isoleucine in that position. Notably, 63 out of the 72 diverse bacterial species represented in the *mrdA* protein family model in the TIGRFAM database (TIGR03423) contain a branched chain amino acid (I, L, or V) at that position, while only three contain a polar residue [45]. Although elucidating the precise mechanism would require further investigation, it is plausible that this mutation is a major contributor to the enhanced halotolerance of strain ht030b.

A thiamine pyrophosphate-binding protein with putative acetolactate synthase activity was found with a mutation in ht030b. Acetolactate synthase catalyzes the first step in the synthesis of branched-chain amino acids [46]. A study of cells from diverse origins carried out by Sévin et al*.* found a negative correlation between halotolerance and the intracellular concentration of branched chain amino acids [47]. Amino acids and amino acid derivatives can be accumulated as compatible solutes as a mechanism of halotolerance [48]. Therefore, changes to enzymes involved in the synthesis of amino acids could divert metabolic fluxes leading to the synthesis of these compatible solutes, although the exact mechanism of this is difficult to predict.

The histidine kinase gene (*NtrY*) mutated in the ALE is predicted to regulate nitrogen metabolism and the assimilation of nitrate [49, 50]. The NtrXY system and the related NtrBC system have been shown in other species to regulate the production and degradation of nitrogen-containing compounds such as arginine and ectoine [51, 52], which again may affect osmotolerance due to the regulation of compatible solutes. The final gene determined to have been altered through the ALE was a transposase of the IS66 family. However, no significant transposition was determined in our variant analysis. In short, several of the unique mutations accumulated by strain ht030b through ALE could conceivably impart halotolerance, although the precise mechanisms are unclear and outside the scope of the present study.

### Deletion of *mscL* gene from *C. necator* enhances cell lysis

A putative *mscL* gene was identified in the *C. necator* genome by a protein BLAST search of the homologous gene found in *E. coli*. The entire gene was deleted from *C. necator* H16 and successful *ΔmscL* mutants were screened by colony PCR and verified by Sanger Sequencing. Deletion of the *mscL* gene led to no obvious deleterious effects on the microbe, besides the desired sensitivity to osmotic downshock. Growth curves of both wild-type and mutant strains were obtained under identical conditions (Additional file 1: Fig. S2). The growth rate of the mutant H16 *ΔmscL* (0.43 ± 0.01 h^−1^) was not statistically different from the growth rate of wild-type H16 (0.45 ± 0.01 h^−1^), indicating that the genome deletion did not impact overall cellular fitness.

Both wild-type H16 and the mutant H16 *ΔmscL* were transformed with pBADT-rfp, containing the RFP gene under an inducible arabinose promoter, and the effect of the *mscL* gene deletion on the fraction of cells lysed upon osmotic downshock was tested. LB was supplemented with NaCl such that the final concentration of NaCl in the medium was 1.5% (w/v). This was the highest salt concentration at which the wild-type *C. necator* H16 strain still displayed measurable overnight growth (Additional file 1: Fig. S3), maximizing the possible magnitude of osmotic downshock while still enabling functional cell growth. As described in the methods section in detail, RFP was expressed in both strains overnight, and washed cells were resuspended either in distilled water or an aqueous solution of 0.5%, 1%, or 1.5% (w/v) NaCl. After 30-min incubations, cells were pelleted and the ratio of red fluorescence intensity found in the supernatant to the fluorescence intensity of the whole solution was taken to be the osmolysis efficiency, or the fraction of cells lysed due to osmotic downshock.

For both wild-type and knockout strains, the highest osmolysis efficiency was observed when cells were resuspended in distilled water, as this caused the highest magnitude of osmotic downshock (0.51 OsM) and therefore the highest osmotic pressure (1.3 MPa). However, significantly greater cell lysis efficiencies were achieved with the *mscL* knockout strain (62%) compared to the wild-type (19%). This demonstrates that the native function of the putative *mscL* gene in *C. necator* is involved in the cell survival response following osmolarity changes, as it is in other bacteria. Following downshock, most of the cells in the wild-type *C. necator* sample remain intact, whereas a majority of cells are lysed when the gene is deleted. Deletion of this gene, therefore, is a simple strategy to increase the susceptibility of microbial hosts and aid in the recovery of intracellular biomolecules.

Similar levels of background cell lysis, which we define as cell lysis observed when resuspended in an isotonic solution, are observed in both the *mscL*^+^ and *ΔmscL* strains (< 5%). Therefore, the *mscL* gene knockout does not make *C. necator* significantly more fragile under normal conditions. The increase in cell lysis only occurs upon osmotic downshock. This, along with the lack of change in the growth rate in H16 *ΔmscL* mentioned previously, suggests that the *mscL* gene is not critical to *C. necator* survival under normal conditions.

This experiment was repeated in defined medium using sodium formate as a sole carbon and energy source to evaluate osmolysis following autotrophic growth. Although there is interest in using *C. necator* for bioproduction under heterotrophic conditions [9, 53], autotrophic production of biomolecules using molecules such as formic acid or hydrogen gas as energy sources enables electromicrobial production. *C. necator* was grown in M9 mineral medium with 4 g/L sodium formate as a carbon source with variable concentrations of added sodium chloride. The maximum amount of NaCl that could be added to M9 medium was determined to be 6 g/L (Additional file 1: Fig. S3). The osmolarity of the medium with this much added salt (0.49 OsM) is close to the osmolarity of LB with 1.5% NaCl (0.51 OsM). Nearly identical trends were seen in experiments with LB and M9 formate, with both H16 and H16 *ΔmscL* experiencing greater osmolytic efficiencies as the magnitude of osmotic downshock increased. The mutant strain lysed significantly more than the wild-type strain (60% vs 18%) when resuspended in distilled water, similar to the LB experiment. While not surprising, as the *mscL* gene is not known to affect cellular metabolism and therefore the effect of its absence should be indifferent towards the carbon metabolism used, this does demonstrate that this strategy can be used to aid downstream recovery for both heterotrophic and autotrophic processes.

### Osmolysis efficiency of *C. necator* ht030b and ht030b *ΔmscL*

**Fig. 3:**
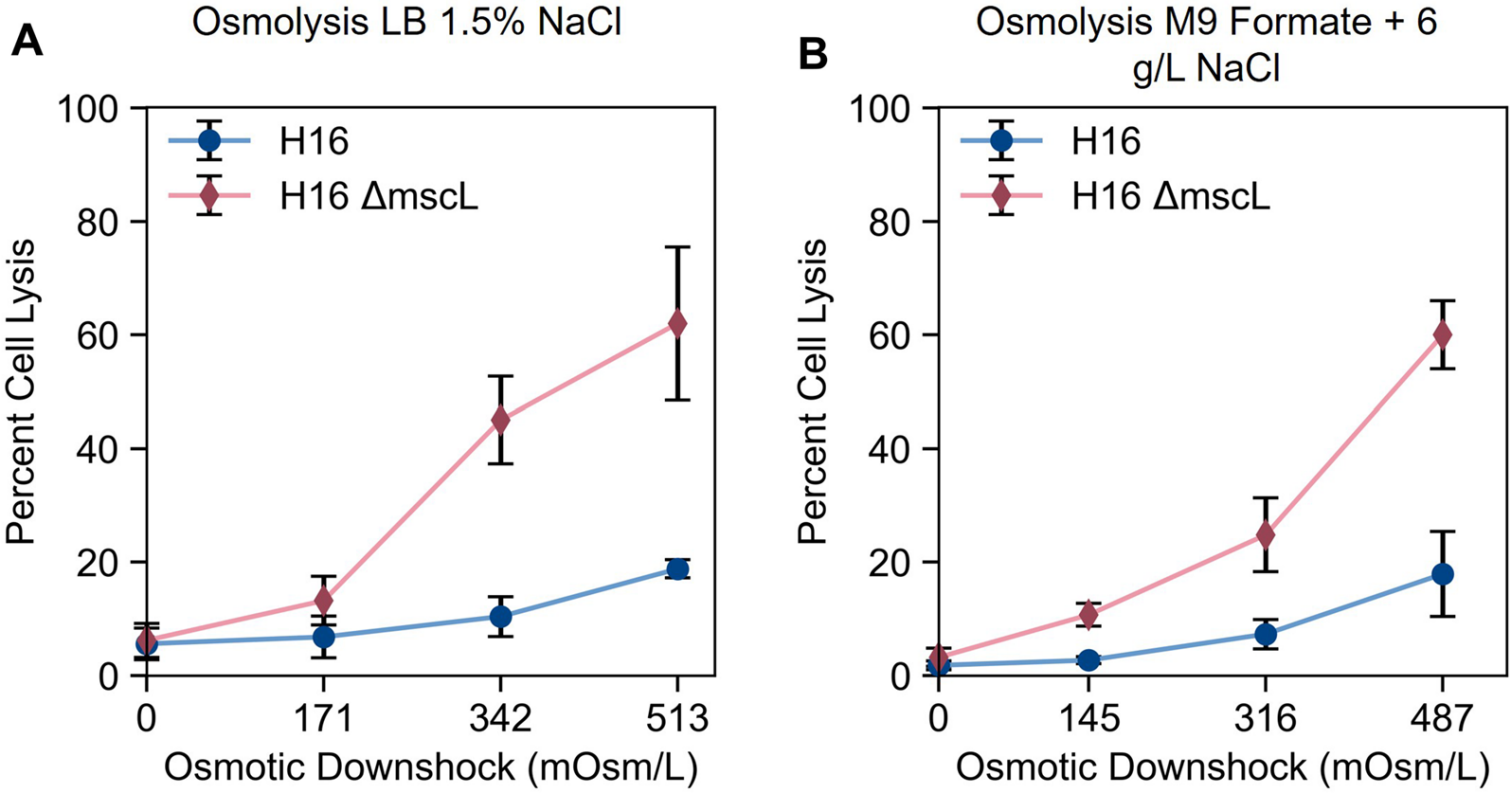
Osmolysis efficiency, calculated as a fraction of RFP recovered in supernatant compared to total RFP content following osmotic shock (see methods), as a function of osmotic downshock magnitude for wild-type *C. necator* H16 (blue circles) and mutant *C. necator* H16 *ΔmscL* (red diamonds) following growth in (**A**) LB with 1.5% (w/v) NaCl (final concentration) and (**B**) M9 sodium formate (4 g/L) medium supplemented with 6 g/L NaCl. Differences in values on the x-axis of the two graphs reflect slight differences in starting osmolarities of the two media tested. For each data point, mean ± SD is displayed (n = 6)

**Fig. 4:**
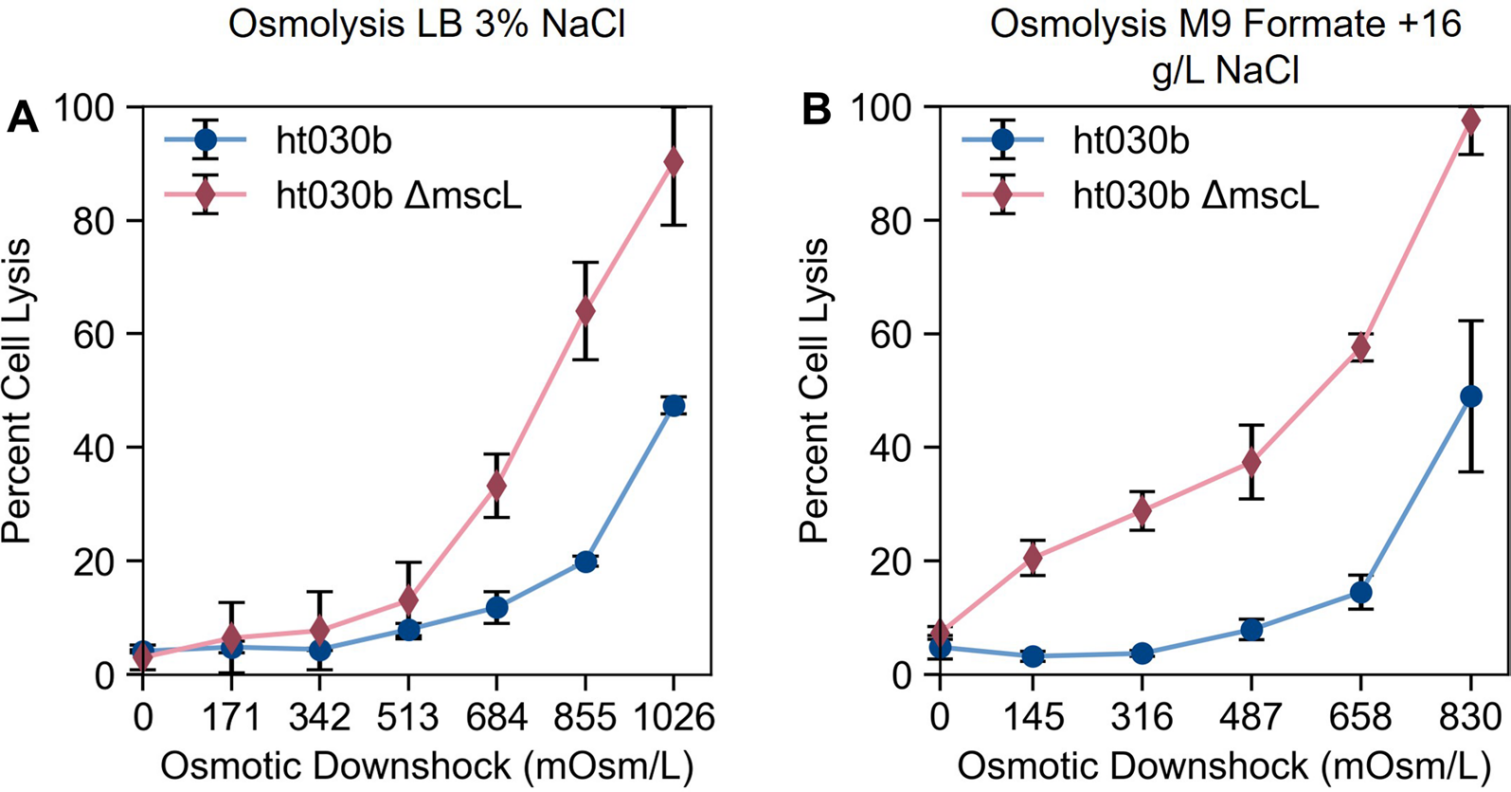
Effect of combined *mscL* gene knockout and improved halotolerance on osmolysis in *C. necator*. Osmolysis efficiency, calculated as a fraction of RFP recovered in cell-free supernatant compared to total RFP content following osmotic shock (relative concentrations determined by fluorescence intensity), as a function of osmotic downshock magnitude for ht030b (blue circles) and ht030b *ΔmscL* (red diamonds) following growth in (**A**) LB with 3.0% NaCl (w/v, final concentration) and (**B**) M9 sodium formate (4 g/L) medium supplemented with 16 g/L NaCl. Differences in values on the x-axis of the two graphs reflect differences in starting osmolarities of the two media tested. For each data point, mean ± SD is displayed (n = 6)

The evolved halotolerant strain of *C. necator* (labeled ht030b) was transformed with the plasmid pBADTrfp and osmolysis was tested following growth in LB supplemented with 3% NaCl (w/v, final concentration) following the same fluorescence-based lysis assay used in the previous section for *C. necator* H16 and H16 *ΔmscL.* Cells were resuspended in aqueous solutions ranging from 0% (distilled water) to 3% NaCl in 0.5% increments and lysis efficiency was measured as before (Fig. 4A). As expected, the maximum cell lysis (47%) was observed after resuspension in distilled water, corresponding to an osmotic pressure change of 1.03 OsM. This is more than double the lysis efficiency observed when resuspending wild-type *C. necator* H16 in distilled water (19%, Fig. 3A). Adapting bacteria for growth in higher salinities allows for higher levels of osmolysis. Therefore, both strategies described in Fig. 1, adapting the microbial host to greater halotolerance and deleting the large-conductance mechanosensitive channel gene, led to enhanced cell lysis in *C. necator*.

These two strategies were then combined in a single strain by deleting the *mscL* gene from the evolved ht030b strain. Successful gene deletion was confirmed by colony PCR and the resultant strain was transformed with the RFP-expressing plasmid. The experiment performed on ht030b was then performed on this new strain. As with the unevolved *C. necator*, deletion of the *mscL* gene significantly enhanced the fraction of cells lysed, with over 90% osmolysis efficiency observed when ht030b *ΔmscL* was resuspended in distilled water (Fig. 4). The combination of the two strategies described led to a greater osmolysis efficiency than either strategy independently. While the osmolysis efficiency of wild-type *C. necator* was only 19% at its maximum, nearly complete lysis of ht030b *ΔmscL* was achieved (compare Figs. 3A and 4A). The combination of ALE to increase halotolerance and the gene deletion of mechanosensitive channels is clearly an effective method for engineering osmolytic susceptibility in a microbial host, which can greatly simplify downstream bioprocessing.

Interestingly, the magnitude of osmotic downshock, and therefore the magnitude of osmotic pressure, does not alone determine the cell lysis efficiency. For both *mscL*^+^ and *ΔmscL* strains, significantly greater cell lysis occurred upon moving cells from 1.5% NaCl_(aq)_ to distilled water (19% for *mscL*^+^ and 62% for *ΔmscL*) than from 3.0% to 1.5% NaCl_(aq)_ (8% for *mscL*^+^ and 13% for *ΔmscL*), despite equivalent osmotic pressure changes in each scenario. Similar observations can be made in the experiments with formate media. The salinity of the resuspension media also plays a role in determining the efficiency of cell lysis. It’s possible that certain membrane proteins take on different conformations in deionized water than they do under normal salt concentrations. Therefore, the cells would experience greater cell lysis in distilled water despite the same osmotic pressure change.

While *C. necator* ht030b grew moderately well in 3% NaCl under heterotrophic conditions (in LB), it did not grow as well at equivalent salinities during organoautotrophic growth. When M9 formate was supplemented with various concentrations of NaCl, *C. necator* ht030b did not significantly grow when the added NaCl concentration exceeded 16 g/L (Additional file 1: Fig. S3). The total osmolarity of this medium was 830 mOsm/L, which is equivalent in ionic strength to a roughly 2.4% (w/v) NaCl solution. Moderate halotolerance is usually effected by the accumulation of compatible solutes, including sugars and amino acids (and their derivatives), inside the cell to balance osmotic pressure [48]. Rich media such as LB contain an abundance of amino acids, which can easily be imported by the cell and used as compatible solutes directly or converted to compatible solutes. Therefore, it is not particularly surprising to see slight differences in halotolerance in the two media tested.

Strains ht030b and ht030b *ΔmscL* were grown in M9 formate supplemented with 16 g/L NaCl, and the RFP lysis assay was performed. Various solutions ranging from distilled water to 2.4% NaCl were tested to measure cell lysis in response to various magnitudes of osmotic downshock. As in all other experiments, the deletion of the *mscL* gene leads to significantly greater osmolysis efficiencies, reaching 98% of ht030b *ΔmscL* cells compared to 49% of ht030b cells. These strategies for engineering susceptibility to lysis by osmotic downshock are thus useful not only for heterotroph-based bioprocesses, but autotrophic processes as well.

### KO of *mscL* and *mscS *genes in *E. coli* BL21 enables significant protein release

Due to the success of the osmolysis strategy in *C. necator*, we explored whether this technique could be broadly applied to other microbial hosts. *E. coli* BL21, a derivative of *E. coli* B strain deficient in Lon and OmpT proteases routinely used for production of recombinant proteins, was chosen as a second model system to evaluate this strategy. Because *E. coli* BL21 could already grow in elevated NaCl concentration, maintaining around half of its maximum growth rate even in 4% NaCl (Additional file 1: Fig. S5), further adaptation of the strain was unnecessary. Therefore, only the effect of the mechanosensitive channel gene deletions was tested.

**Fig. 5:**
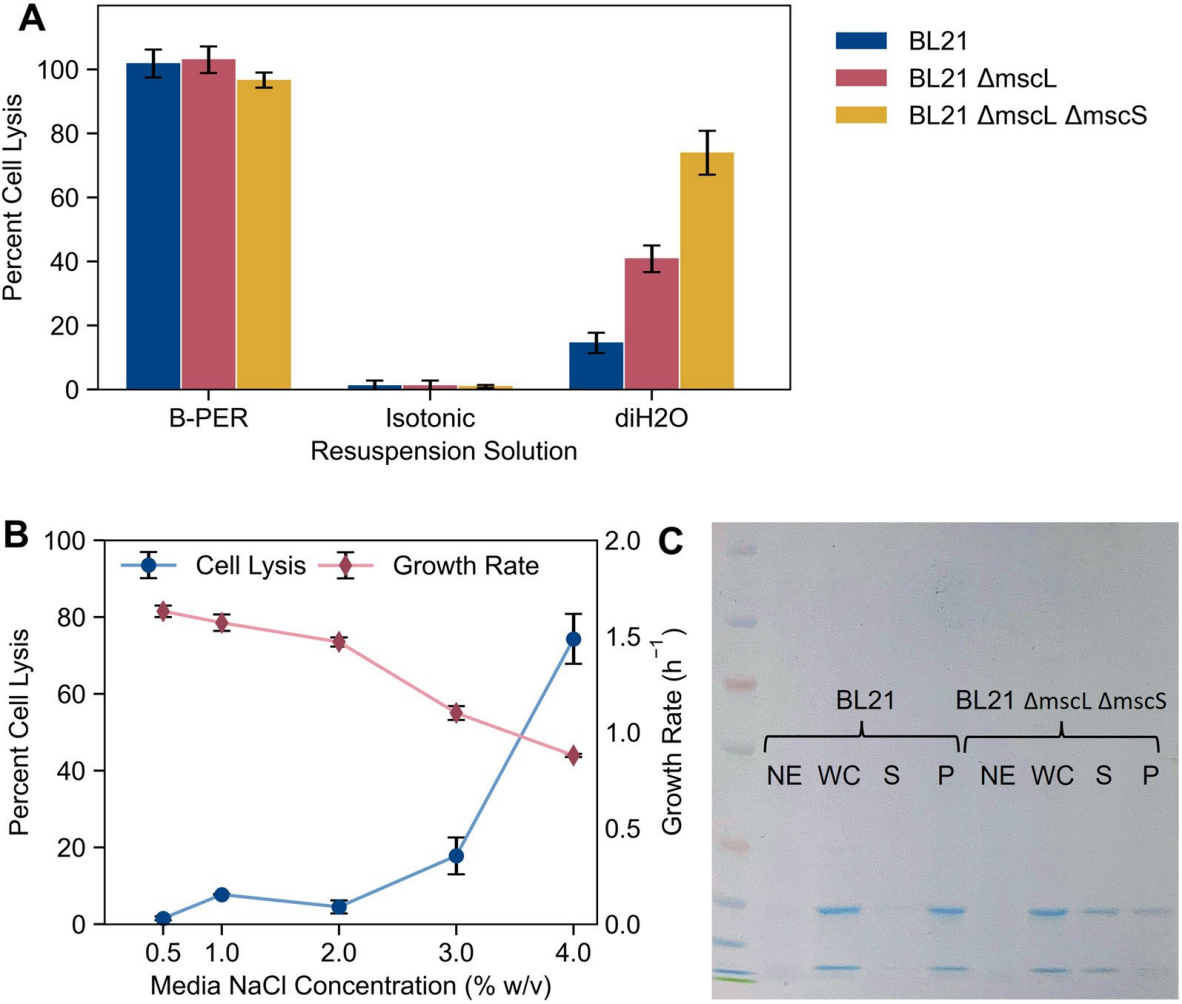
Application of mechanosensitive knockout for osmolysis in *E. coli* BL21. **A** Percent cell lysis following growth in LB with 4% NaCl of BL21 (blue), BL21 *ΔmscL* (red), and BL21 *ΔmscL ΔmscS* (yellow) in three different media: commercial B-PER™ Bacterial Protein Extraction Reagent; a 4% NaCl_(aq)_ isotonic solution; and distilled water (diH2O). Cell lysis is determined as the fraction of RFP recovered in supernatant compared to total RFP content, as it was in *C. necator*, with concentration measured by fluorescence intensity (n = 6). **B** Growth rate (red diamonds) and osmolysis efficiency (blue circles) of BL21 *ΔmscL ΔmscS* in LB with various (final) NaCl concentrations (n = 3). **C** SDS-PAGE gel of various fractions of RFP-expressing BL21 and BL21 *ΔmscL ΔmscS.* NE: cells not expressing RFP, WC: whole-cell fraction, S: post-osmolysis supernatant fraction, P: post-osmolysis cell pellet. For each data point in (**A**) and (**B**), mean ± SD is displayed

The *mscL* gene was successfully knocked out of *E. coli* BL21, as confirmed by colony PCR, and the resultant strain was transformed with the RFP-expressing plasmid pBADTrfp. The RFP-based osmolysis assay was then performed as it was for *C. necator* (with minor variations, see methods for details), comparing the engineered and wild-type strains. Cultures of these strains were grown in LB containing 4% (w/v) NaCl and cell lysis was tested following resuspension in distilled water, 4% NaCl isotonic aqueous solution, or B-PER^™^, a commercial bacterial lysis reagent (ThermoFisher Scientific) used as a positive control. As it did in *C. necator*, deleting the *mscL* gene from *E. coli* BL21 significantly increased the lysis efficiency in distilled water (41% v. 15%). Although this is encouraging evidence that this strategy is broadly applicable in many bacterial hosts, ~ 40% recovery of a given macromolecule product is likely too low in most practical applications. To improve the lysis efficiency, a second gene in the mechanosensitive channel family, the small-conductance mechanosensitive channel (*mscS*) gene, was also deleted from BL21. The double knockout demonstrated increased sensitivity to osmotic shock, reaching an average cell lysis efficiency of 75% (and efficiency as high as 81% in individual trials) following growth in LB with 4% NaCl (Fig. 5A). While a majority of the biomolecule product RFP is separated from the cell biomass through osmolysis, the fractional recovery of the product will be moderately lower than when using detergent-based lysis reagents such as B-PER, which consistently demonstrated near 100% efficiency.

Following the RFP-based assay, SDS-PAGE was performed on the whole-cell, supernatant, and cell pellet fractions post-osmolysis (Fig. 5C). Although more difficult to quantify exactly, the results of the gel are roughly consistent with the results of the RFP assay. Almost no protein is observed in the supernatant for the wild-type BL21 cells following resuspension in distilled water, while a significant fraction is for BL21 *ΔmscL ΔmscS* cells. It appears that a greater fraction of the total protein content is present in the supernatant fraction compared to the cell pellet fraction in the double knockout cells, consistent with the results of the RFP assay. Taken with RFP data, this demonstrates that the BL21 *ΔmscL ΔmscS* strain can be used to greatly simplify the recovery of expressed proteins, while maintaining a high protein recovery.

This osmolysis efficiency is slightly lower than the > 90% cell death rate observed by Levina et al*.* in a double knockout *ΔmscL ΔmscS* strain of *E. coli* K-12 derivative Frag1 following similar osmotic downshock [34]. This difference could be due to the different assays used to measure the effects of osmotic downshock. Levina et al*.* measured cell viability by counting colony-forming units on agar plates while we measure release of intracellular protein. It is possible that more than 75% of cells become non-viable following downshock, yet their membranes remain intact enough to retain the cytosolic contents.

To test the effect of media salt concentration on osmolysis efficiency, this experiment was repeated in LB with NaCl concentrations of 0.5%, 1%, 2%, and 3% (w/v, Fig. 5B). We note that 0.5% and 1% NaCl concentration are the two salt concentrations found in most LB formulations. Even in the double knockout strain, little more than background levels of cell lysis were observed for NaCl concentrations of 2% or less. Low (18%), but statistically significant (p < 0.013), levels of cell lysis were observed for BL21 *ΔmscL ΔmscS* beginning at a media NaCl concentration of 3%. A sharp increase in the cell lysis efficiency occurred with an increase from 3 to 4% NaCl, suggesting the critical osmotic pressure required for cell lysis for most cells in the population falls within that range. As expected, the mutant *E. coli* strain experienced the greatest degree of cell lysis upon osmotic downshock following growth in the media with the highest osmolarity tested (4% NaCl). However, under these conditions, the growth rate of *E. coli* BL21 was significantly affected. We observed a roughly linear decrease in specific growth rate of BL21 with increasing NaCl concentrations, a trend demonstrated previously [54]. Increasing the salt concentration in the media from 0.5% to 4% w/v NaCl coincided with a growth rate decrease from 1.6 h^−1^ to 0.88 h^−1^.

In practical applications, a tradeoff would be encountered in which greater product recovery via osmolysis would come at the cost of a slower microbial growth rate. However, for many applications, a decreased growth rate may be worthwhile. The specific growth rate of 0.88 h^−1^ corresponds to a doubling time of roughly 45 min, which is still significantly faster than many microbial hosts. Routine lab-scale protein expression and purification protocols could still be performed in a single day, even at this diminished growth rate, as a seed culture inoculated to an optical density of A_600_ = 0.05 could reach mid-log phase (A_600_ = 0.5) in around 3 h. In continuous industrial-scale reactors, productivity is often limited by oxygenation rate rather than specific growth rate [55]. Therefore, continuous bioreactor systems can likely operate at higher salt concentrations with a similar productivity despite the decrease in growth rate.

Although only a 75% cell lysis efficiency (on average) was attained in our experiments, this efficiency could likely be improved. Slightly higher NaCl concentrations in the media would likely be tolerated by BL21, and if the trend in Fig. 5B continues, one would expect near complete cell lysis could be achieved. Furthermore, this strain could also be used in conjunction with other cell lysis methods. For example, adding a single freeze–thaw step to the process increased the osmolysis efficiency of BL21 *ΔmscL ΔmscS* grown in LB with 2% NaCl from 4.5% to 22% (Additional file 1: Fig. S6), while such an increase was not observed for BL21 without the gene deletions. In summary, the engineered BL21 variant becomes more susceptible to cell lysis in distilled water than the original BL21 strain, which can be exploited for simplified protein purification.

## Conclusion

Although downstream bioseparations represent a critical (and costly) component of any biochemical process, most genetic engineering and strain development has been performed to aid in the generation of a product rather than its purification. Focusing on cell lysis as an important step in purification of intracellular bioproducts, we present strategies that utilize adaptive laboratory evolution and rational genome engineering to produce strains that are sensitive to osmotic downshock, and therefore may be used for simplified, osmolysis-based downstream recovery. First, we have shown that *Cupriavidus necator* can be made slightly halotolerant relatively quickly through adaptive laboratory evolution. The evolved strain ht030b was the result of 30 passages of ALE, which improved its halotolerance from 15 to 32.5 g/L NaCl. This adaptation alone improved the maximum efficiency of osmolysis from 19 to 47%. A halotolerant strain of *C. necator* also provides benefits in other areas of electromicrobial production. In particular, we have recently shown through reactor modelling that, for certain products such as lactic acid produced through EMP, effects of salinity-induced toxicity can limit the productivity of bioproduction systems [23]. Therefore, the halotolerant strain of *C. necator* evolved here could be useful in improving productivity of systems limited by salinity effects.

In parallel, a strain of *C. necator* was engineered by deleting the *mscL* gene, which increased the cell lysis efficiency following osmotic downshock from 19 to 62%. While the putative function of this gene in *C. necator* was inferred by homology to be a large-conductance mechanosensitive channel, we provide the first experimental evidence of its function. We then combined the two strategies in a single strain, *C. necator* ht030b *ΔmscL*, which exhibited the highest cell lysis efficiency of over 90% when grown in LB medium and 99% when grown in M9 formate, demonstrating for the first time the efficacy of combining adaptive laboratory evolution with rational mechanosensitive channel knockouts to enhance cell lysis efficiency.

We then adapted these techniques to develop a strain of *E. coli* BL21, a common strain for protein expression, that is susceptible to osmolysis. Two gene knockouts (*mscL* and *mscS*) were required for significant (75%) protein release, though adaptive laboratory evolution was unnecessary. Previous experiments focusing on the *mscL* and *mscS* genes, which have included knockouts, were done primarily to study the function of these genes. Therefore, phenomena such as cell viability have been studied rather than biomacromolecule release. We provide, to our knowledge, the first study of mechanosensitive channel knockouts to aid the recovery of intracellular macromolecules.

We have demonstrated that the implementation of our strategies leads to significant cell lysis in both *E. coli* and *C. necator*, allowing intracellular bioproducts to be easily recovered from the strains, using red fluorescent protein as a model product. Either of the strategies described here for increasing osmolysis should be broadly applicable, as many (especially non-marine) bacteria contain the *mscL* gene [33], and adaptive laboratory evolution is suitable for most bacterial strains. Whether this general approach is also applicable to gram-positive bacteria or archaea given their different cell membrane structures [56] remains an interesting avenue for future exploration, although previous research suggests *mscL* deletions in gram-positive *B. subtilis* causes increased cell death upon osmotic downshock [57].

We have also shown that *C. necator* is much more sensitive to osmotic downshock compared to *E. coli*. This is interesting as *C. necator* and *E. coli* are both gram-negative proteobacteria and therefore have similar membrane structures. Under similar osmotic downshocks (equivalent to 3% NaCl) the wild-type *C. necator* strain had a lysis efficiency of 47% compared to only about 3% for *E. coli* cells. This large disparity was also seen among the *ΔmscL* versions of each species, with *C. necator* experiencing 90% cell lysis and *E. coli* experiencing only 14% cell lysis following a 3% (w/v) NaCl osmotic downshock (Additional file 1: Fig. S7). Therefore, although we have shown that knocking out mechanosensitive channel genes does increase osmolytic sensitivity in both strains, the cell lysis efficiencies can vary from strain to strain. This strategy must therefore be tested in individual strains to assess the viability of the osmolysis technique.

While the cell lysis efficiencies obtained through osmolysis were quite high (90% for *C. necator* and 75% for *E. coli* BL21), other established methods of cell lysis can achieve higher values. For example, we have demonstrated that detergent-based methods routinely achieve near total cell lysis (Fig. 5A), while Sauer et al. showed that high-pressure homogenization leads to similarly high (95–98%) cell lysis efficiencies [58]. However, some methods of cell lysis, such as repeated freeze–thaw cycles are less effective (~ 50% protein recovery) [59]. We also note that all of the values mentioned will depend on the bacterial strain, scale of operation, and other conditions, making direct comparison to other methods in the literature subject to some uncertainty.

Whether cell lysis fractions obtained through osmolysis are sufficiently high for a given bioprocess will depend on a number of factors, including the product. It is likely that low-value products such as biopolymers would be more suitable for osmolysis compared to high-value products such as therapeutic proteins. If the product of interest is very valuable, then the cost savings of the simplified cell lysis method will likely not outweigh the marginal gain of value from using more expensive but more effective cell lysis methods. Therefore, the efficacy of osmolysis will depend on the specific application, including the strain used and biomolecule produced. Economic and environmental impact concerns should also be taken into consideration in determining whether osmolysis or a traditional cell lysis method is used prior to downstream product recovery. Nonetheless, we have conclusively demonstrated two broadly applicable strategies to improving the susceptibility of bacteria to osmolysis that are both independently effective and compatible with each other in a single strain. These results serve as a foundation for potentially simpler and cheaper downstream biomolecule processing, an area often overlooked in fundamental research.

## Supplementary Information

**Additional file 1: Table S1.** Mutations Detected in H16 and ht030b strains; **Figure S1.** Growth curve of H16 (blue circles) and ht030b (red diamonds) in LB containing 3.25% NaCl in 50-mL cultures in shake flasks, seeded at an optical density of A_600nm_ = 0.05. **Figure S2.** Growth Curve of *C. necator* H16 (blue circles) and H16 *ΔmscL* (red diamonds); **Figure S3.** Measured optical densities of *C. necator* H16 (A) and *C. necator* ht030b (B) following 24 h of growth in LB at various salt (final) concentrations as well as H16 (C) and ht030b (D) following 48 h of growth in M9 formate with various (added) salt concentrations. Black dashed line represents cutoff OD of 0.22 (LB growth) and 0.077 (M9 growth) which defines thresholds of salt tolerance in the respective media. **Figure S4.** Overview of RFP-based cell lysis assay developed. (A) Schematic overview of RFP assay as described in methods. Well-mixed red fluorescence measurements (585 nm excitation/ 620 nm emission) were performed on the well-mixed sample, representing the total RFP content, and from the supernatant following centrifugation, representing the released RFP content. Cell lysis fraction was taken to be the ratio of released RFP to total RFP. (B) Representative linear range validation that was replicated in each experiment to verify that RFP concentration was proportional to fluorescence intensity. (C) Fluorescence intensity measurements of identical RFP-expressing cell samples in various solutions, demonstrating that the fluorescence intensity is not sensitive to the various environments encountered in the assay. **Figure S5.** (A) Growth of *E. coli* BL21 at various salt concentrations as a function of time. (B) Semilog of cell density as a function of time during logarithmic growth phase. **Figure S6.** Effect of addition of freeze–thaw step (yellow) with osmolysis for BL21 and BL21 *ΔmscL ΔmscS* compared to cells only subjected to osmotic downshock (blue). **Figure S7.** Percent cell lysis of *E. coli* BL21 (blue), BL21 *ΔmscL* (red), and BL21 *ΔmscL ΔmscS* (yellow) in three different media: commercial B-PER™ Bacterial Protein Extraction Reagent, a 3% NaCl_(aq)_ isotonic solution, and distilled water. (n = 3).

## Data Availability

The genomic datasets generated and analyzed during the current study are available in the Sequence Read Archive (https://www.ncbi.nlm.nih.gov/bioproject/906984). All other data generated or analyzed during this study are included in this published article and its supplementary information files.
