# Supplementary material for: Engineering osmolysis susceptibility in Cupriavidus necator and Escherichia coli for recovery of intracellular products
Source: Microb Cell Fact. 2023 Apr 12;22:69. doi: 10.1186/s12934-023-02064-8 (PMC10091555; doi:10.1186/s12934-023-02064-8)
Supplement: Supplementary file 1 — Additional file 1: Table S1. Mutations Detected in H16 and ht030b strains; Figure S1. Growth curve of H16 (blue circles) and ht030b (red diamonds) in LB containing 3.25% NaCl in 50-mL cultures in shake flasks, seeded at an optical density of A600nm = 0.05. Figure S2. Growth Curve of C. necator H16 (blue circles) and H16 ΔmscL (red diamonds); Figure S3. Measured optical densities of C. necator H16 (A) and C. necator ht030b (B) following 24 h of growth in LB at various salt (final) concentrations as well as H16 (C) and ht030b (D) following 48 h of growth in M9 formate with various (added) salt concentrations. Black dashed line represents cutoff OD of 0.22 (LB growth) and 0.077 (M9 growth) which defines thresholds of salt tolerance in the respective media. Figure S4. Overview of RFP-based cell lysis assay developed. (A) Schematic overview of RFP assay as described in methods. Well-mixed red fluorescence measurements (585 nm excitation/ 620 nm emission) were performed on the well-mixed sample, representing the total RFP content, and from the supernatant following centrifugation, representing the released RFP content. Cell lysis fraction was taken to be the ratio of released RFP to total RFP. (B) Representative linear range validation that was replicated in each experiment to verify that RFP concentration was proportional to fluorescence intensity. (C) Fluorescence intensity measurements of identical RFP-expressing cell samples in various solutions, demonstrating that the fluorescence intensity is not sensitive to the various environments encountered in the assay. Figure S5. (A) Growth of E. coli BL21 at various salt concentrations as a function of time. (B) Semilog of cell density as a function of time during logarithmic growth phase. Figure S6. Effect of addition of freeze–thaw step (yellow) with osmolysis for BL21 and BL21 ΔmscL ΔmscS compared to cells only subjected to osmotic downshock (blue). Figure S7. Percent cell lysis of E. coli BL21 (blue), BL21 ΔmscL (red), a [file 12934_2023_2064_MOESM1_ESM.pdf]

Supplementary Information

Engineering osmolysis susceptibility in *Cupriavidus necator* and *Escherichia coli* for recovery of intracellular products

Jeremy David Adams[1], Kyle B. Sander[2], Craig S. Criddle[3], Adam P. Arkin[2,4], Douglas S. Clark[1,5*]

Affiliations
1. Department of Chemical and Biomolecular Engineering, University of California, Berkeley, Berkeley, CA 94720, USA
2. Department of Bioengineering, University of California, Berkeley, Berkeley, CA 94720, USA
3. Department of Civil and Environmental Engineering, Stanford University, Stanford, CA 94305, USA
4. Environmental Genomics and Systems Biology Division, Lawrence Berkeley National Laboratory, 1 Cyclotron Road, Berkeley, CA 94720, USA
5. Molecular Biophysics and Integrated Bioimaging Division, Lawrence Berkeley National Laboratory, 1 Cyclotron Road, Berkeley, CA 94720, USA

[*]Correspondence should be addressed to D.S.C (dsc@berkeley.edu)

Supplementary Tables

Table S1: Mutations Detected in H16 and ht030b strains

**Chromosome (NCBI Accession No.)**	**Genome Coordinates**	**Gene/ Encoded Protein**	**Mutation (CDS position)**	**Effect**	**SNP Present In***
1 (CP039287)	3982709	PAS domain-containing sensor histidine kinase	G→A (1606)	Substitution (R→C)	ht030b
1 (CP039287)	131230	*mrdA* Peptidoglycan D,D-transpeptidase	C→T (1688)	Substitution (T→I)	ht030b
1 (CP039287)	791503	*YgcG* family protein	C→A (757)	Substitution (G→W)	H16
2 (CP039288)	829590	Acetolactate synthase	C→T (1490)	Substitution (T→I)	ht030b
pHG-1 (CP039289)	138984	IS66 family transposase	T→A (206)	Substitution (E→V)	ht030b

*Whole genome sequences of both the parent strain H16 and the evolved strain ht030b were obtained to identify the mutations that arose throughout the ALE. Both genomes were first mapped to the reference *C. necator* H16 genome obtained by Little *et al*,[1] and differences between each of the two genomes we sequenced and the reference genomes were identified. Several of these variations were found in both the parent strain and evolved strain, indicating these mutations were not acquired throughout the ALE. Five variations (meeting quality control criteria described in the Methods) were unique to either the parent or evolved strain. Four variations (relative to the reference genome) were found in ht030b, while one was found in the unevolved H16 strain. This is denoted by the column labeled "SNP Present In".

Supplementary Note 1: Growth of H16 and ht030b in high salt media

Figure 2B in the main text compares the growth of wild-type *C. necator* H16 with the adapted halotolerant strain ht030b. In that experiment, 4 replicate cultures each of H16 and ht030b were grown in a 24-well plate and grown overnight at 30 °C, with starting optical densities (A_{600nm}) of 0.01. H16 demonstrated no visible growth, whereas ht030b exhibited exponential growth with a specific growth rate of 0.16 h^{-1}. However, we have found that growth of H16 in high salt conditions appears dependent on the starting optical density of the culture and other culturing conditions. A similar experiment was therefore performed in 50-mL volumes in 250 mL baffled shake flasks. Both H16 and ht030b were seeded to starting optical densities of ~0.05.

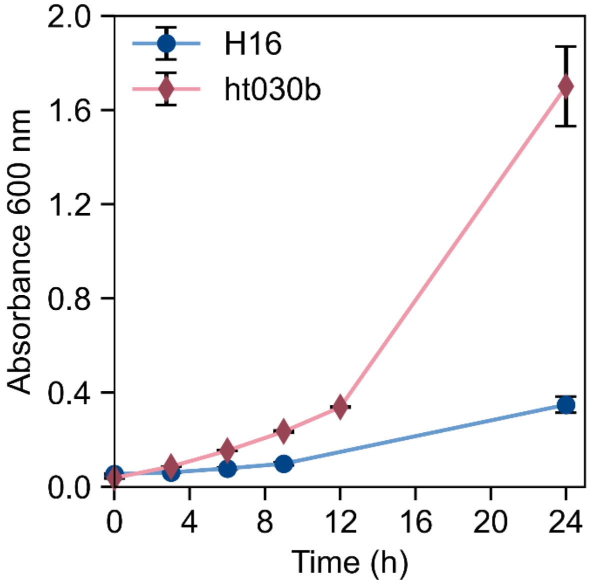

Fig. S1 Growth curve of H16 (blue circles) and ht030b (red diamonds) in LB containing 3.25% NaCl in 50-mL cultures in shake flasks, seeded at an optical density of A_{600nm}=0.05.

Although wild-type H16 did grow slightly in LB containing 32.5 g/L NaCl (final concentration) in this experiment, the evolved strain still grew significantly faster. Calculated specific growth rates were 0.18 h^{-1} for ht030b and 0.08 h^{-1} for H16. In addition to the higher starting cell concentration, it is likely that greater oxygen mass transfer was achieved in flasks compared to that in 24-well plates. The growth of H16 is somewhat dependent on the culturing conditions when growing in LB at elevated salt concentrations. However, in all cases, the evolved strain ht030b grew significantly better than the wild-type strain in high salt concentrations.

Supplementary Note 2: Growth of Wild-type and *mscL* knockout *Cupriavidus necator*

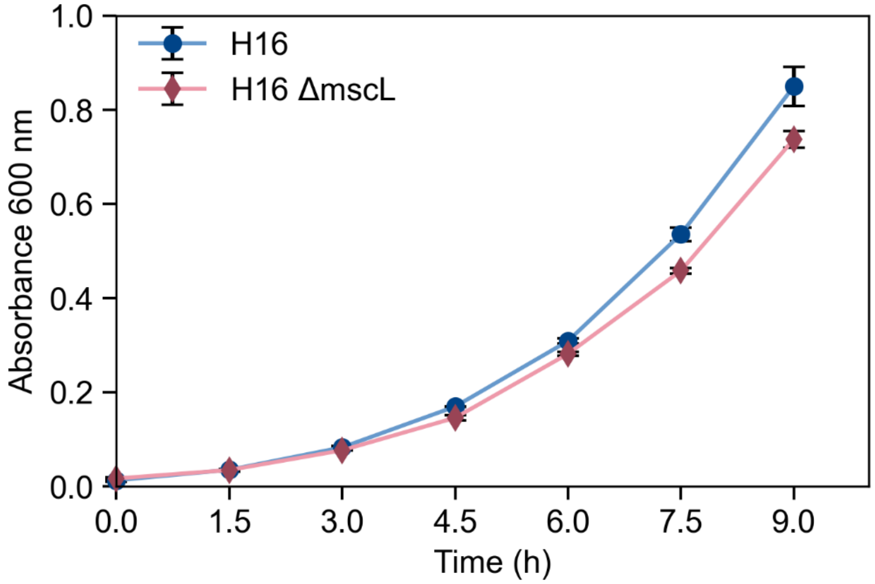

Fig. S2 Growth Curve of *C. necator* H16 (blue circles) and H16 *ΔmscL* (red diamonds)

Growth curves were measured for both wild-type *Cupriavidus necator* H16 and *C. necator ΔmscL* in LB medium. Overnight cultures of both strains were inoculated to an initial cell density with A_{600}=0.015 in 50-mL cultures in shake flasks. Cultures were grown at 30 ºC shaking at 200 rpm for 9 hours, with absorbance measurements (600 nm) taken every 90 minutes.

The growth curves of the two strains in LB were not significantly different. The measured growth rate of the wild-type strain (0.45 ± 0.01 h^{-1}) was just slightly higher than the growth rate of the *ΔmscL* strain (0.43 ± 0.01 h^{-1}). Although there was an observable difference between the growth of the two strains, this result was not statistically significant ($p>0.07$). Therefore, we conclude that the absence of the *mscL* gene does not significantly affect the growth rate of *C. necator*, and that the *mscL* gene is not required for normal functioning of the cell.

Supplementary Note 3: Effect of salt concentration on growth of *C. necator* H16 and *C. necator* ht030b in LB and M9 Formate

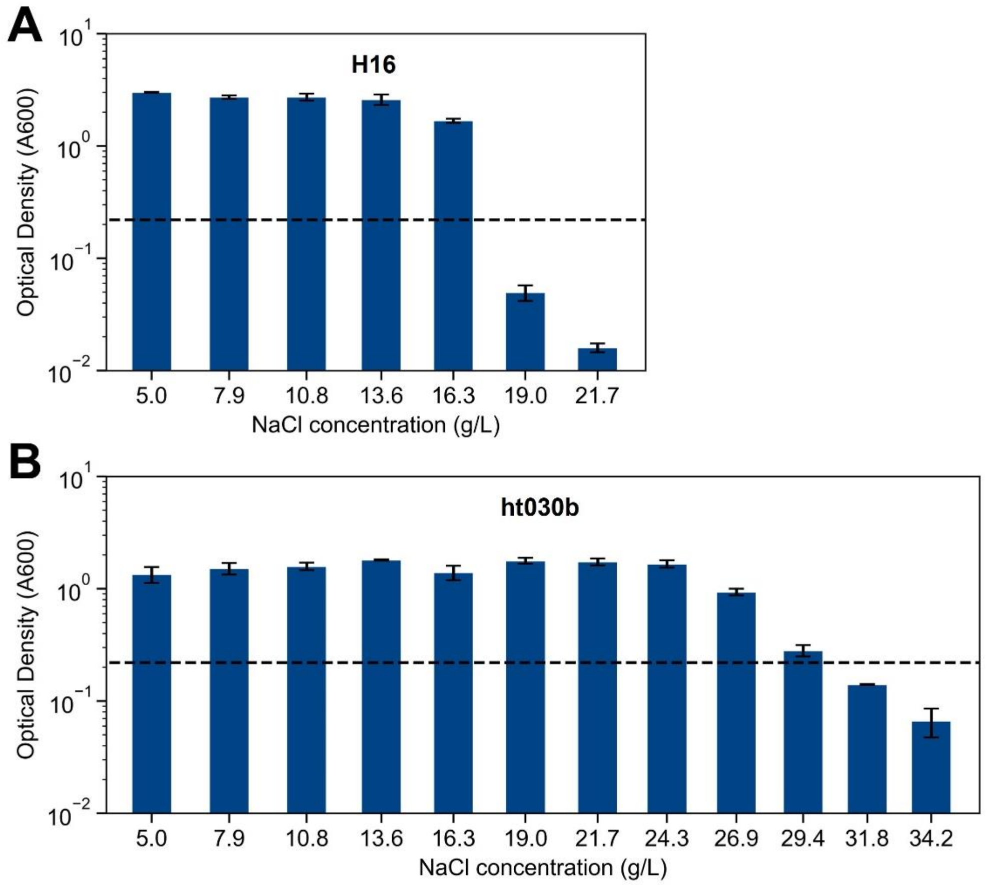

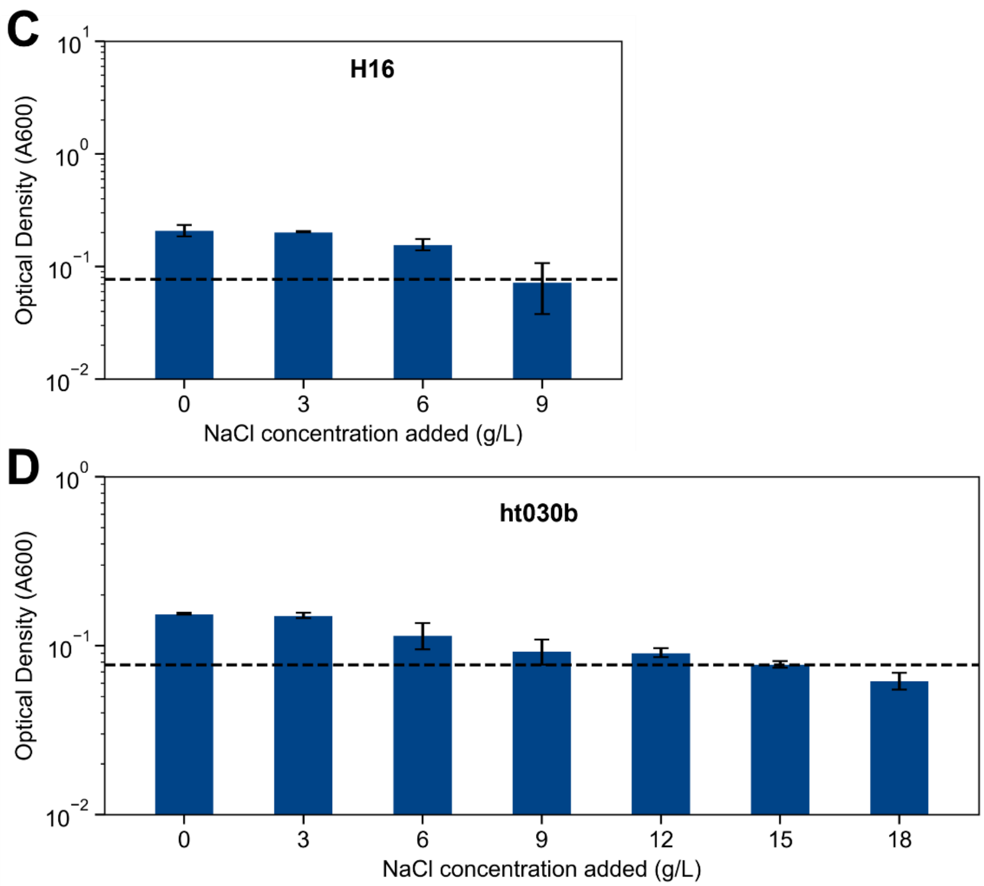

Fig. S3. Measured optical densities of *C. necator* H16 (A) and *C. necator* ht030b (B) following 24 hours of growth in LB at various salt (final) concentrations as well as H16 (C) and ht030b (D) following 48 hours of growth in M9 formate with various (added) salt concentrations. Black dashed line represents cutoff OD of 0.22 (LB growth) and 0.077 (M9 growth) which defines thresholds of salt tolerance in the respective media.

The maximum salt concentration tolerated by both wild-type *C. necator* H16 and evolved strain ht030b was determined for both heterotrophic growth (LB) and organoautotrophic growth (M9 formate). To test salt tolerance for heterotrophic growth, both strains were inoculated in 50-mL tubes containing 10 mL LB with variable salt concentrations to a starting OD of 0.001. As the measured average growth rate of *C. necator* H16 was 0.45 h^{-1}, we defined the salt tolerance as the maximum salt concentration for which the average growth rate over a 24-hour period exceeded 0.225 h^{-1} (half of normal growth rate). This corresponded to an optical density of over 0.22 after a 24-hour period.

The NaCl concentrations tolerated by H16 and ht030b were 16.3 and 29.4 g/L, respectively. For convenience, NaCl concentrations of 15 g/L and 30 g/L were used for H16 and ht030b respectively for osmolysis experiments of those two strains.

To test NaCl tolerance under formatotrophic growth, various salt concentrations were added to M9 formate (note: these represent the amount of salt *added* to M9 medium, which already contains various amounts of certain salts, rather than the final salt concentration; final osmolarities are taken

into account in the data shown in Figures 3B and 4B in the main text). Both strains (H16 and ht030b) were then inoculated in 50-mL tubes containing 10 mL of formate media to a starting OD of 0.02. Formatotrophic growth in defined medium was significantly slower than heterotrophic growth in rich medium. Optical densities were measured after 48 hours. The optical density threshold for maximum tolerated salt concentration was 0.077, which is half of the measured OD of ht030b after 48 hours in M9 formate with no added salt.

The NaCl concentrations tolerated by H16 and ht030b in M9 formate were 6 g/L and 15 g/L respectively. Therefore, M9 formate with 6 g/L added was used as the growth medium for the experiments described in Figure 3B. For the experiments described in Figure 4B, M9 formate with 16 g/L was used. As shown in Fig. S3D, the drop in cell growth when the added salt concentration is raised from 15 g/L to 18 g/L is fairly small. M9 formate with 16 g/L NaCl added has an osmolarity of 0.834 OsM, which is roughly equivalent to that of a 2.5% NaCl solution. Because osmolysis experiments were performed with salt solutions in 0.5% (w/v) increments, this was a more convenient starting solution from a practical standpoint.

Supplementary Note 4: RFP-Based Cell Lysis Assay Diagram and Measurement Notes

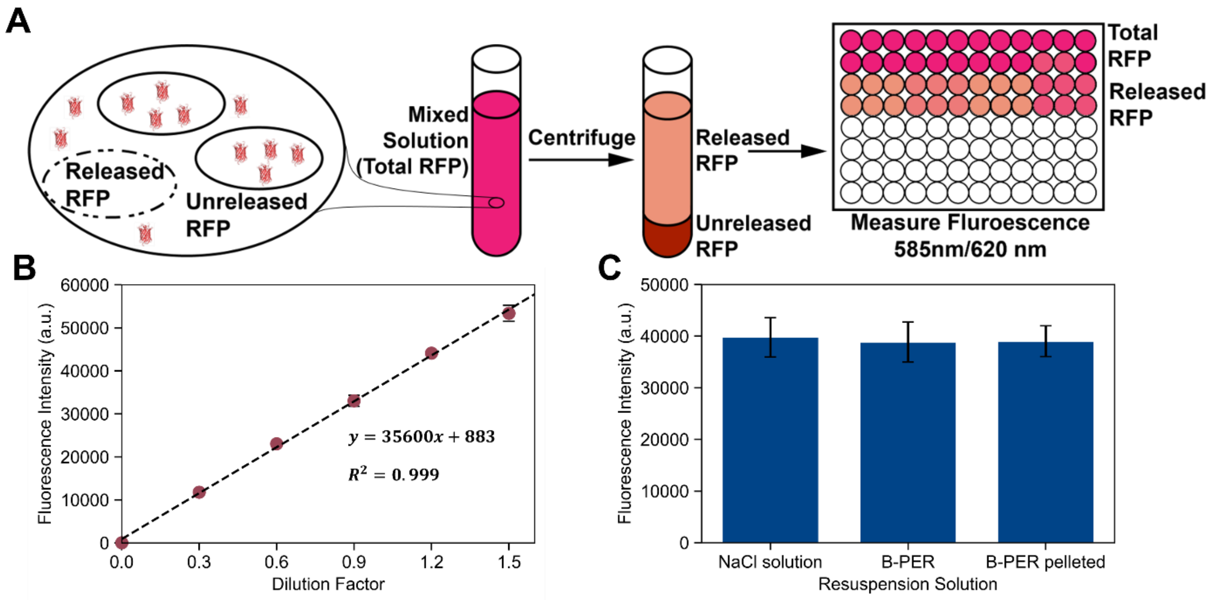

Fig S4. Overview of RFP-based cell lysis assay developed. (A) Schematic overview of RFP assay as described in methods. Well-mixed red fluorescence measurements (585 nm excitation/ 620 nm emission) were performed on the well-mixed sample, representing the total RFP content, and from the supernatant following centrifugation, representing the released RFP content. Cell lysis fraction was taken to be the ratio of released RFP to total RFP. (B) Representative linear range validation that was replicated in each experiment to verify that RFP concentration was proportional to fluorescence intensity. (C) Fluorescence intensity measurements of identical RFP-expressing cell samples in various solutions, demonstrating that the fluorescence intensity is not sensitive to the various environments encountered in the assay.

In each osmolysis experiment relying on the RFP-based cell lysis assay described in the main text, samples were verified to ensure they fell within the linear range. Cells expressing RFP following the wash step in the osmolysis protocol were concentrated or diluted such that they were 30%, 60%, 90%, 120%, or 150% of the original cell density. Volumes equivalent to the volume measured in the experiment (usually 150 µL for experiments using *C. necator* and 50 µL for experiments using *E. coli*) were aliquoted into a 96-well plate and red fluorescence was measured (same excitation/emission values as described in main text methods). If the standard curve was linear, and all samples measured fell within the linear range, then the osmolysis measurements were considered valid. A representative standard curve is shown in Figure S4B. If needed, samples were further diluted in water such that they did fall within this linear range.

Our assay relies on the assumption that the fluorescent signal is a function only of the concentration of RFP in the sample (*i.e.*, that neither the solvent nor the presence/absence of cells significantly affects the fluorescence measurement). To verify this was always the case, fluorescence measurements were taken on three types of samples encountered throughout the experiments. All

samples were prepared from equal volumes of the same culture, and therefore began with same amount of RFP. One sample was resuspended in an aqueous salt solution, and therefore nearly all of the RFP remained within the cell. One sample was resuspended in B-PER™ (a commercial bacterial lysis reagent) and therefore cell membranes were lysed and nearly all the RFP was in solution. In the final sample, cells were resuspended in B-PER™ but were then centrifuged, such that RFP was present in a supernatant free of cell debris. As seen in Fig. S4C, all three samples have nearly identical fluorescence values, within 3% of each other. Therefore, we are confident in assuming that neither the solvent nor the location of RFP with respect to cell biomass significantly impacts fluorescence measurement, and therefore our assay is valid in comparing RFP concentration in the various fractions.

Supplementary Note 5: Growth of BL21 *ΔmscL ΔmscS* in various salt concentrations

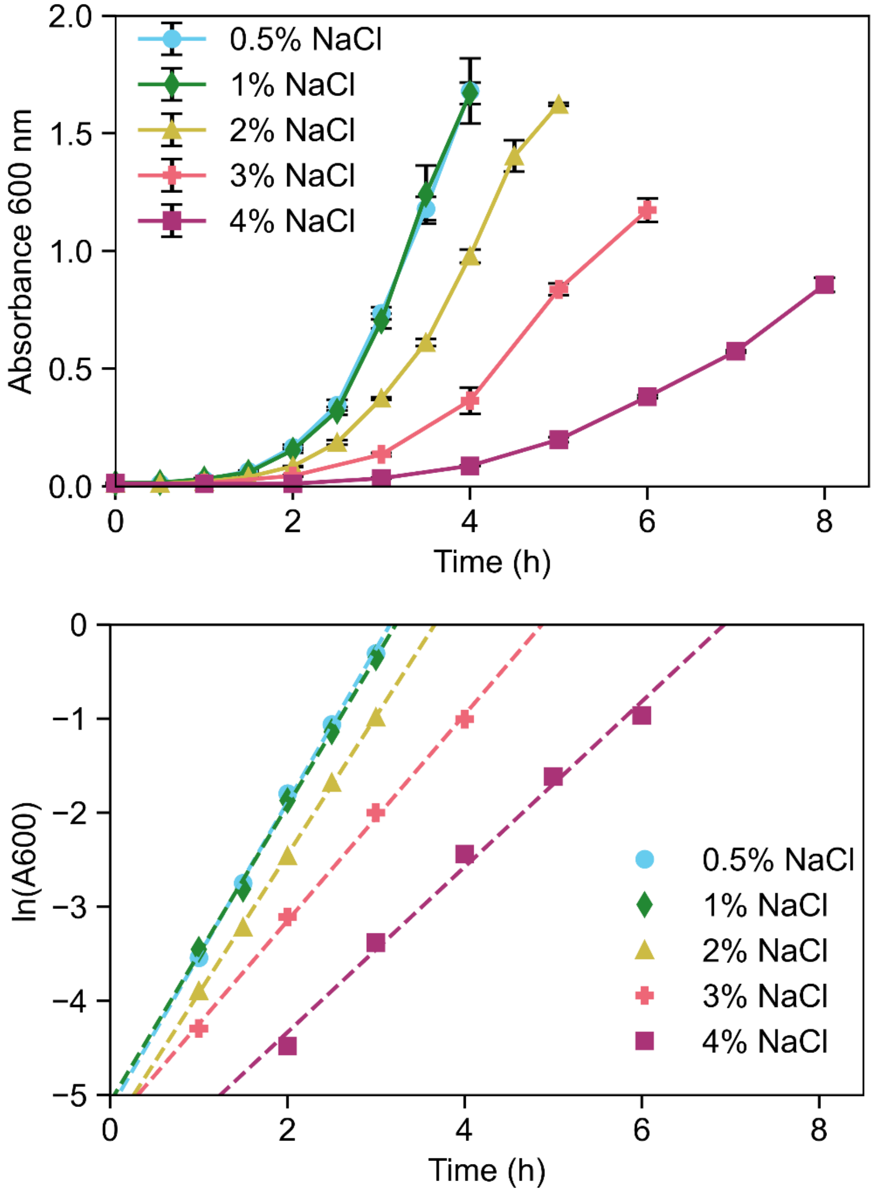

Fig. S5: (A) Growth of *E. coli* BL21 at various salt concentrations as a function of time. (B) Semilog of cell density as a function of time during logarithmic growth phase.

As described in the main text, the growth rate of *E. coli* BL21 *ΔmscL ΔmscS* was measured to demonstrate a tradeoff between the microbial growth rate and osmolysis efficiency. Growth curves were determined for this strain in LB supplemented with NaCl (if necessary) to final concentrations of 0.5%, 1%, 2%, 3%, and 4% (w/v). Cultures were grown in 50-mL volumes in 250-mL baffled shake flasks at 37 ºC, starting at an optical density of 0.01. Absorbance measurements were taken every half hour for cultures grown in 0.5%, 1%, and 2% salt and every hour for cultures grown in 3% and 4% salt. Specific growth rates were calculated from the slope

of the line of a semilog plot for the range in which the log of absorbance was linear with respect to time.

Supplementary Note 6: Cell Lysis Combined with Freeze-thaw enhances cell lysis

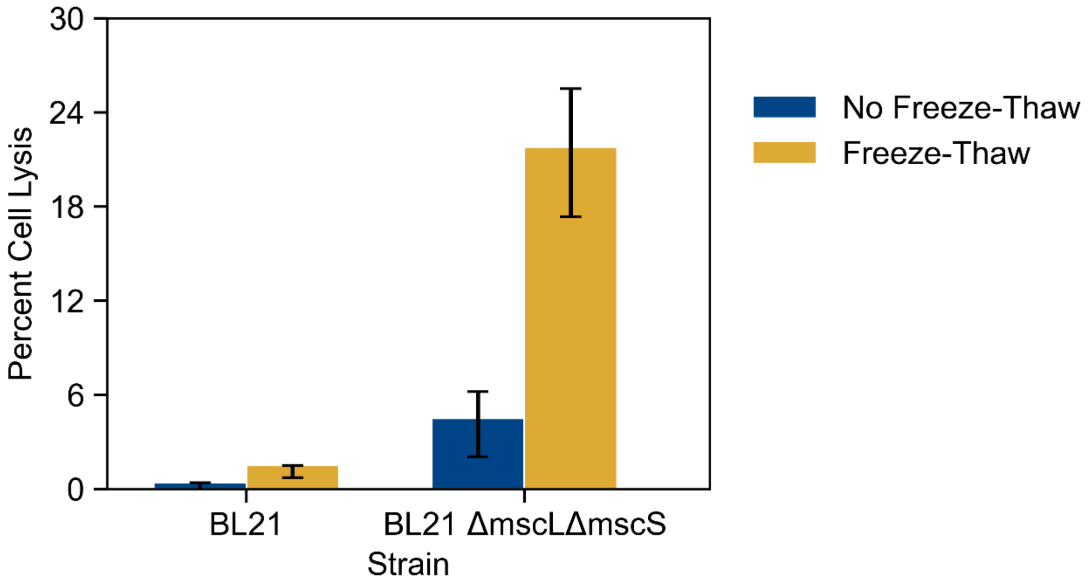

Fig. S6 Effect of addition of freeze-thaw step (yellow) with osmolysis for BL21 and BL21 *ΔmscL ΔmscS* compared to cells only subjected to osmotic downshock (blue).

The effect the adding a freeze-thaw step to osmolysis was determined for BL21 cells grown in LB with 2% NaCl (w/v). The procedure was the same as for other osmolysis experiments with minor modifications. Cells were grown, RFP was expressed, and cells were washed as they were in other BL21 osmolysis experiments. For trials labelled "No Freeze-Thaw" samples were resuspended in distilled water and incubated for 30 min at 30 ºC as was normally done. For samples treated with a freeze-thaw step, however, cells were resuspended in distilled water, placed in a freezer set at −20 ºC for twenty minutes, and then thawed in a heat block set at 37 ºC for ten minutes. Samples from the well-mixed culture and supernatant were taken and measured as they were in previous experiments.

Adding a freeze-thaw step significantly enhances the cell lysis efficiency in BL21 *ΔmscL ΔmscS* cells. The highest cell lysis (22%) is observed for BL21 *ΔmscL ΔmscS* cells that are subjected to freeze-thaw, which is roughly 5-fold higher than lysis of BL21 *ΔmscL ΔmscS* without a freeze-thaw step and 15-fold higher than lysis of BL21 with a freeze-thaw. This improvement indicates that even higher cell lysis efficiencies may be obtained by combining osmolysis with other methods of cell lysis.

Supplementary Note 7: Osmolysis of BL21 after growth in 3% NaCl

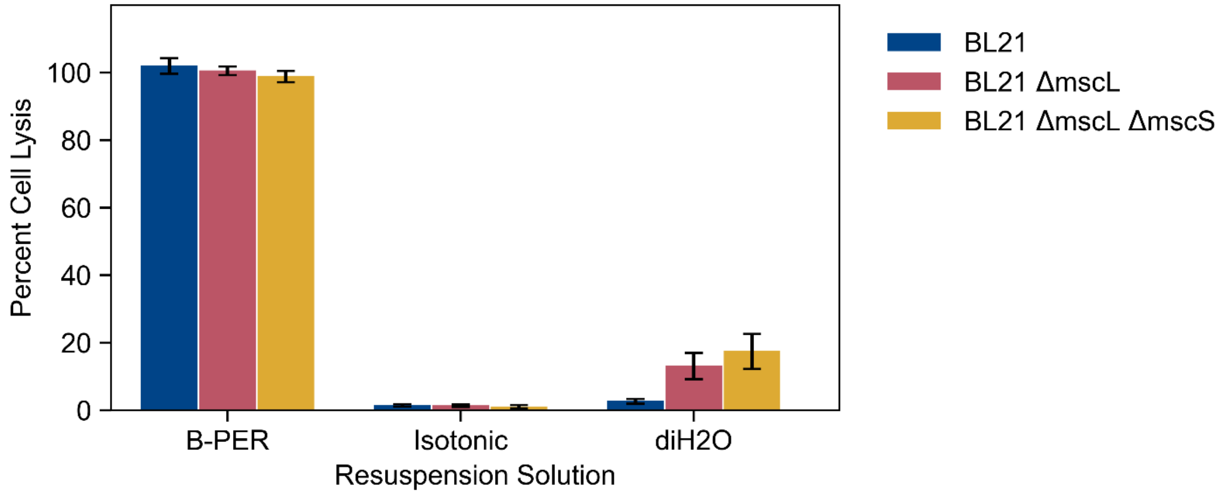

Fig. S7. Percent cell lysis of *E. coli* BL21 (blue), BL21 *ΔmscL* (red), and BL21 *ΔmscL ΔmscS* (yellow) in three different media: commercial B-PER™ Bacterial Protein Extraction Reagent, a 3% $NaCl_{(aq)}$ isotonic solution, and distilled water. (n=3).

Experiments described in Fig. 5A of the main text were repeated exactly, except with cells grown in LB containing 3% NaCl. Note the considerable difference between osmolytic efficiencies of cells grown in 3% and 4% NaCl. This also allows direct comparison of osmolysis between *C. necator* ht030b and BL21 (as well as their *ΔmscL* variants), as they were both grown in 3% NaCl. The percent cell lysis in distilled water following growth in 3% NaCl LB was >90% for ht030b *ΔmscL* and 14% for BL21 *ΔmscL*.

References

1. T., L. G. *et al.* Complete Genome Sequence of Cupriavidus necator H16 (DSM 428). *Microbiol. Resour. Announc.* **8**, e00814-19 (2019).
